# Identification of distinct immune signatures in inclusion body myositis by peripheral blood immunophenotyping using machine learning models

**DOI:** 10.1002/cti2.1504

**Published:** 2024-04-03

**Authors:** Emily McLeish, Anuradha Sooda, Nataliya Slater, Kelly Beer, Ian Cooper, Frank L Mastaglia, Merrilee Needham, Jerome D Coudert

**Affiliations:** ^1^ Centre for Molecular Medicine and Innovative Therapeutics Murdoch University Murdoch WA Australia; ^2^ Perron Institute for Neurological and Translational Science Nedlands WA Australia; ^3^ School of Medicine University of Notre Dame Australia Fremantle WA Australia; ^4^ Department of Neurology Fiona Stanley Hospital Murdoch WA Australia

**Keywords:** AI, IBM, inflammatory myopathies, machine learning, random forest

## Abstract

**Objective:**

Inclusion body myositis (IBM) is a progressive late‐onset muscle disease characterised by preferential weakness of quadriceps femoris and finger flexors, with elusive causes involving immune, degenerative, genetic and age‐related factors. Overlapping with normal muscle ageing makes diagnosis and prognosis problematic.

**Methods:**

We characterised peripheral blood leucocytes in 81 IBM patients and 45 healthy controls using flow cytometry. Using a random forest classifier, we identified immune changes in IBM compared to HC. K‐means clustering and the random forest one‐versus‐rest model classified patients into three immunophenotypic clusters. Functional outcome measures including mTUG, 2MWT, IBM‐FRS, EAT‐10, knee extension and grip strength were assessed across clusters.

**Results:**

The random forest model achieved a 94% AUC ROC with 82.76% specificity and 100% sensitivity. Significant differences were found in IBM patients, including increased CD8^+^ T‐bet^+^ cells, CD4^+^ T cells skewed towards a Th1 phenotype and altered γδ T cell repertoire with a reduced proportion of Vγ9^+^Vδ2^+^ cells. IBM patients formed three clusters: (i) activated and inflammatory CD8^+^ and CD4^+^ T‐cell profile and the highest proportion of anti‐cN1A‐positive patients in cluster 1; (ii) limited inflammation in cluster 2; (iii) highly differentiated, pro‐inflammatory T‐cell profile in cluster 3. Additionally, no significant differences in patients' age and gender were detected between immunophenotype clusters; however, worsening trends were detected with several functional outcomes.

**Conclusion:**

These findings unveil distinct immune profiles in IBM, shedding light on underlying pathological mechanisms for potential immunoregulatory therapeutic development.

## Graphical abstract

Peripheral blood leucocytes from 81 inclusion body myositis (IBM) patients and 45 healthy controls were analysed using flow cytometry. A supervised computational approach using the random forest classifier identified immune signatures in IBM, while unsupervised modelling stratified patients into distinct immunophenotypic clusters. These findings shed light on the underlying pathological mechanisms and hold potential for future clinical applications, spanning IBM diagnosis, prognosis and management.

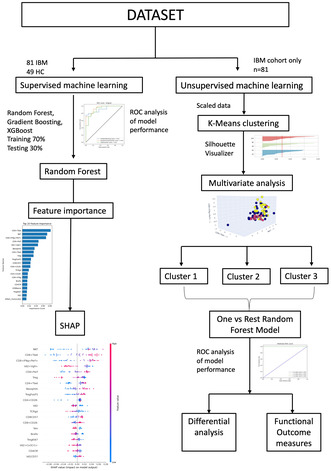

## Introduction

Inclusion body myositis (IBM) is a devastating and progressive late‐onset muscle disease that presents with gradual loss of muscle strength and mass and is poorly responsive to treatment.^1^ It is considered one of the most challenging and complex of all muscle diseases, characterised by a preferential pattern of weakness of proximal muscles such as the quadriceps femoris and hip flexors in the lower limbs and finger and wrist flexors in the upper limbs. As a result, everyday activities such as gripping objects, climbing stairs and rising from a chair become increasingly difficult and there is a propensity to falls.^1^, ^2^ In addition, a high proportion of IBM patients experience dysphagia as a result of the involvement of the bulbar muscles.^3^, ^4^

Despite extensive research efforts, the underlying aetiopathogenesis of IBM remains unclear. The current consensus is that there are multiple contributors, including immune, degenerative, genetic and ageing factors.^5^ Understanding each of these factors and their role in the aetiopathogenesis of IBM is important to progress our understanding of disease and potential therapies. Our work within this study is focused on understanding the underlying immune‐mediated mechanisms of IBM by comprehensively analysing patients' immune cell composition and phenotypic characteristics. Multiple types of immune cells, including T cells, B cells, monocytes, natural killer cells (NK) and dendritic cells (DC), have all been found to play a role in the pathogenesis of IBM.^6^, ^7^, ^8^, ^9^ Previous immunophenotyping studies have reported that CD8^+^ T cells, displaying a terminally differentiated (T_EMRA_) phenotype, also referred to as ‘senescent’, infiltrate the muscle endomysium and invade muscle fibres.^7^ These cells are characterised by a lack of proliferation, resistance to apoptosis and increased effector functions. Additionally, T regulatory cells (Tregs), which are beneficial in autoimmune conditions as a result of their suppressive activity on immune effector cells, are reduced in IBM patients compared to healthy age‐matched controls.^10^ Gamma‐delta (γδ) T cells are an unconventional population of lymphocytes comprising approximately 5% of the T‐cell population.^11^ They possess features of both innate and adaptive immunity and are capable of recognising antigens or tissue damage and eliciting cytotoxic activity in an atypical MHC‐independent manner. Earlier studies identified γδ T cells surrounding and invading nonnecrotic muscle fibres in some cases of polymyositis^12^, ^13^; however, their role in IBM has not been fully elucidated. Additionally, the presence of antibodies directed against cytosolic 5′‐nucleotidase 1A (cN1A) has been detected in a proportion of IBM patients (37–72%),^9^, ^14^, ^15^, ^16^ suggesting that a self‐directed humoral response may play a role in the disease.^17^ IBM patients vary significantly in terms of their clinical presentation and rate of progression.^18^ This is likely due, at least in part, to variable underlying immune changes. As a result, defining the range of immune and pathological alterations that characterise the disease is extremely challenging.

Machine learning (ML) is a rapidly growing field of artificial intelligence involving the development of algorithms that learn patterns in datasets and make predictions or decisions based on that learning. In the context of biomedicine, ML is being used to analyse high‐dimensional and complex data sets to improve diagnostic accuracy and discover new pathways in disease mechanisms.^19^, ^20^ To date, several studies investigating the phenotypical, genomic, metabolomic and transcriptomic alterations in idiopathic inflammatory myopathies (IIM) have utilised the power of ML models.^21^, ^22^, ^23^, ^24^, ^25^ Recent studies have shown that unsupervised clustering models can be used to identify discrete groups of IIM patients based on their immune profiles.^21^, ^26^, ^27^

In this study, we conducted comprehensive immunophenotyping of peripheral blood leucocytes using multi‐parameter flow cytometry. Our analysis encompassed a comparative cross‐sectional exploration of IBM patients and healthy controls. Given IBM prevalence in those aged 50 and above, we strategically compared IBM with similarly aged HC, distinctly isolating disease‐specific immune shifts from age‐related influences. We also aimed to compare immunophenotypes between IBM patients via unsupervised clustering techniques and examine correlations with clinical and functional measures, deepening our comprehension of IBM heterogeneous nature.

## Results

### Cohort demographics and lymphocyte counts

Cohort demographics and absolute counts of the major lymphocyte sub‐populations in peripheral blood are summarised in Table 1. The median age was 74 ± 9.9 years in the IBM group and 68 ± 9.7 years in the HC group (*P‐*value = < 0.001). Spearman's correlation analysis did not reveal any strong correlation between age and immune cell populations in IBM or HC (Supplementary figure 1a, b). There was a higher proportion of males to females in the IBM group than in HCs (*n* = 48:33 vs. *n* = 20:25, respectively) but the difference was not statistically significant (Chi‐square test *P‐*value = 0.16).

**Table 1:** Demographics and peripheral blood leucocyte counts (per L of blood) in IBM patients and healthy controls (HC)

	IBM (*N = 81*)	HC (*N = 45*)	*P*‐value
Age (years)	74 (41–98)	68 (47–87)	< 0.001
Male:Female	48:33	20:25	0.16
Lymphocytes	9.6 × 10^8^ (1.1 × 10^8^–2.7 × 10^9^)	1.1 × 10^9^ (3.27 × 10^8^–2.7 × 10^9^)	0.06
T cells	5.1 × 10^8^ (5.0 × 10^7^–1.8 × 10^9^)	6.2 × 10^8^ (1.87 × 10^8^–1.6 × 10^9^)	< 0.05
CD8^+^ T cells	1.4 × 10^8^ (8.8 × 10^6^–1.0 × 10^9^)	1.3 × 10^8^ (3.89 × 10^7^–6.8 × 10^8^)	0.68
CD4^+^ T cells	3.0 × 10^8^ (2.5 × 10^7^–1.0 × 10^9^)	4.1 × 10^8^ (1.11 × 10^8^–9.3 × 10^8^)	< 0.05
CD4:CD8 ratio	1.9 (0.4–38.7)	2.2 (1.05–12.2)	0.12
B cells	0.97 × 10^8^ (5.1 × 10^6^–5.0 × 10^8^)	1.6 × 10^8^ (4.92 × 10^7^–7.4 × 10^8^)	< 0.001
*Gamma delta T cells	1.5 × 10^7^ (6.7 × 10^5^–1.3 × 10^8^)	1.6 × 10^7^ (6.7 × 10^5^–1.1 × 10^8^)	0.09
*Vδ2^+^	2.0 × 10^6^ (0–3.96 × 10^7^)	8.3 × 10^6^ (1.6 × 10^5^–1.0 × 10^8^)	< 0.001
*Vδ1^+^	4.2 × 10^6^ (7.7 × 10^4^–1.0 × 10^8^)	3.7 × 10^6^ (3.8 × 10^4^–3.1 × 10^8^)	0.89
Vδ2:Vδ1 ratio	0.2 (0–54.2)	1.6 (0.04 28.9)	< 0.0001

All the values are represented as median; the values in parentheses are range. *Absolute counts for gamma delta T cells, Vδ2^+^ and Vδ1^+^ were analysed in 40 IBM patients and 33 healthy controls.

The absolute cell counts of total lymphocytes and CD8^+^ T cells were similar in both groups; however, there was a 25% reduction in CD4^+^ T cell counts in IBM patients compared to HC (0.3 × 10^9^ L^−1^ vs. 0.4 × 10^9^ L^−1^, respectively; *P‐*value < 0.05) (Table 1). Despite this reduction, there was no significant difference in the CD4:CD8 ratios between the groups (*P‐*value = 0.12, Table 1) but we did observe an expansion of the CD8^+^ T cells accompanied by altered CD4:CD8 ratios, below or at a value of 1.5, in 33% of IBM patients and 20% of HC (Supplementary figure 2). The absolute numbers of gamma‐delta (γδ) T cells and frequency distribution of this subset were investigated in 40 IBM patients and 33 HC. Total γδ T cell counts were similar in IBM and HC groups (1.59 × 10^7^ L^−1^ vs. 1.64 × 10^7^ L^−1^, respectively; *P‐*value = 0.09), but there was a significant reduction of the Vδ2 subset in IBM (2.00 × 10^6^ L^−1^ vs. 8.32 × 10^6^ L^−1^ in HC; *P‐*value < 0.001). We also noted that IBM patients had significantly reduced numbers of circulating B cells than HC (0.97 × 10^8^ L^−1^ vs. 1.65 × 10^8^ L^−1^, respectively; *P‐*value ≤ 0.001).

### Distinct blood immunophenotype profiles differentiate IBM patients from healthy controls

**Figure 1:**
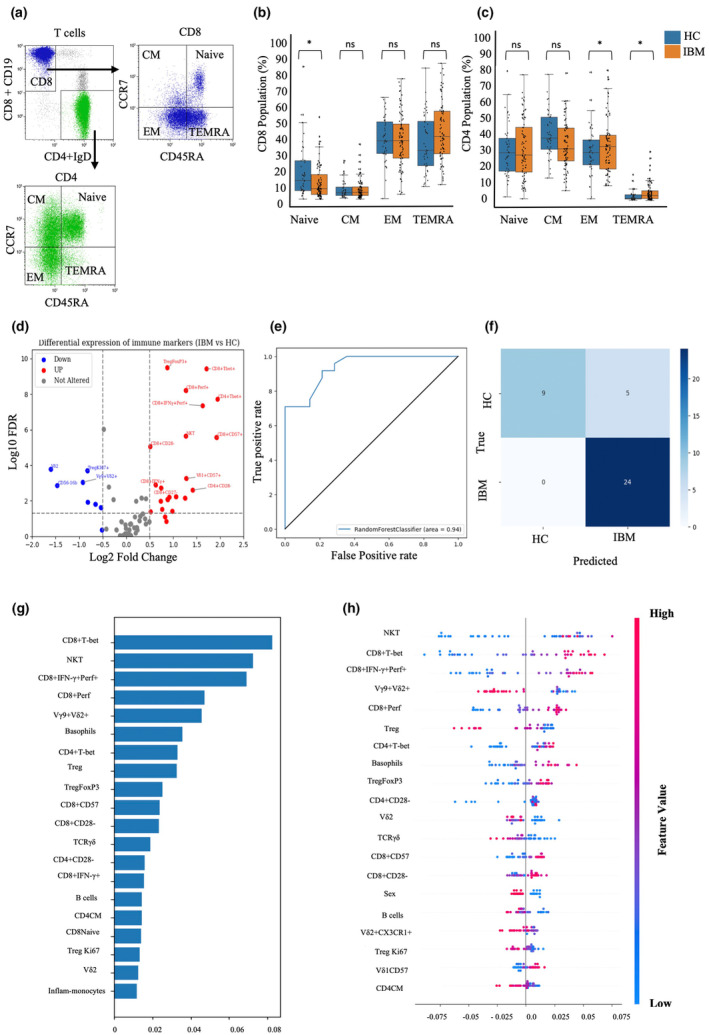
Immunological profile comparison between IBM patients HC. **(a)** Representative flow cytometry biplot showing the gating strategy for memory CD4^+^ and CD8^+^ T cell‐populations. Bar plots show the median values for the percentage of CD8^+^ **(b)** and CD4^+^ **(c)** T cell naïve and memory subsets in HC (*n* = 45) and IBM (*n* = 81). The statistical analysis was performed using the Mann–Whitney *U‐*test. Data are presented as median ± interquartile range. ns = not significant, *P‐value < 0.05. **(d)** Volcano plot showing the differential frequency of cell subsets in IBM patients relative to HC. The *x*‐axis shows the log_2_ fold change (FC) values and the *y*‐axis shows the negative log_10_ transformed *P‐*values (−log_10_ (*P‐*value)). The red dots represent cell subsets that are significantly elevated in IBM patients (log_2_ FC > 0.5) with a false discovery rate (FDR) of 0.05. The blue dots represent cell subsets that are significantly decreased in IBM patients (log_2_ FC < −0.5) with an FDR of 0.05. The black dots represent cell subsets that are not significantly altered in IBM. The vertical dashed lines represent the log_2_ FC cut‐offs (−0.5 and 0.5) while the horizontal dashed line represents the FDR cut‐off (−log_10_ (*P‐*value)) as the defined significance threshold. **(e)** Receiver operator characteristic (ROC) curve illustrating the area under the curve (AUC) for the random forest model applied to the IBM (*n* = 81) and HC (*n* = 49) dataset. The ROC curve visually represents the model performance in distinguishing between the IBM and HC subjects based on the given features, with the AUC indicating the overall accuracy of the model's predictions. **(f)** Confusion matrix illustrating the predicted (*x*‐axis) versus true numbers (*y*‐axis) of IBM and HC subjects obtained from the test data of a random forest model. The matrix provides an assessment of the model's accuracy in correctly classifying subjects into the IBM and HC categories based on the given features. **(g)** Top 20 feature importance plot for the discrimination of IBM from HC calculated using the mean decrease impurity method. **(h)** The local explanation summary, indicating the direction of the relationship between a variable and disease outcome. Each dot on the plot represents the SHAP value of a variable for a single participant, with its position along the *x*‐axis indicating whether the contribution was additive or subtractive for that participant. The colour of each dot represents the value of the corresponding variable, with high positive values shown in red and low negative values shown in blue.

**Figure 2:**
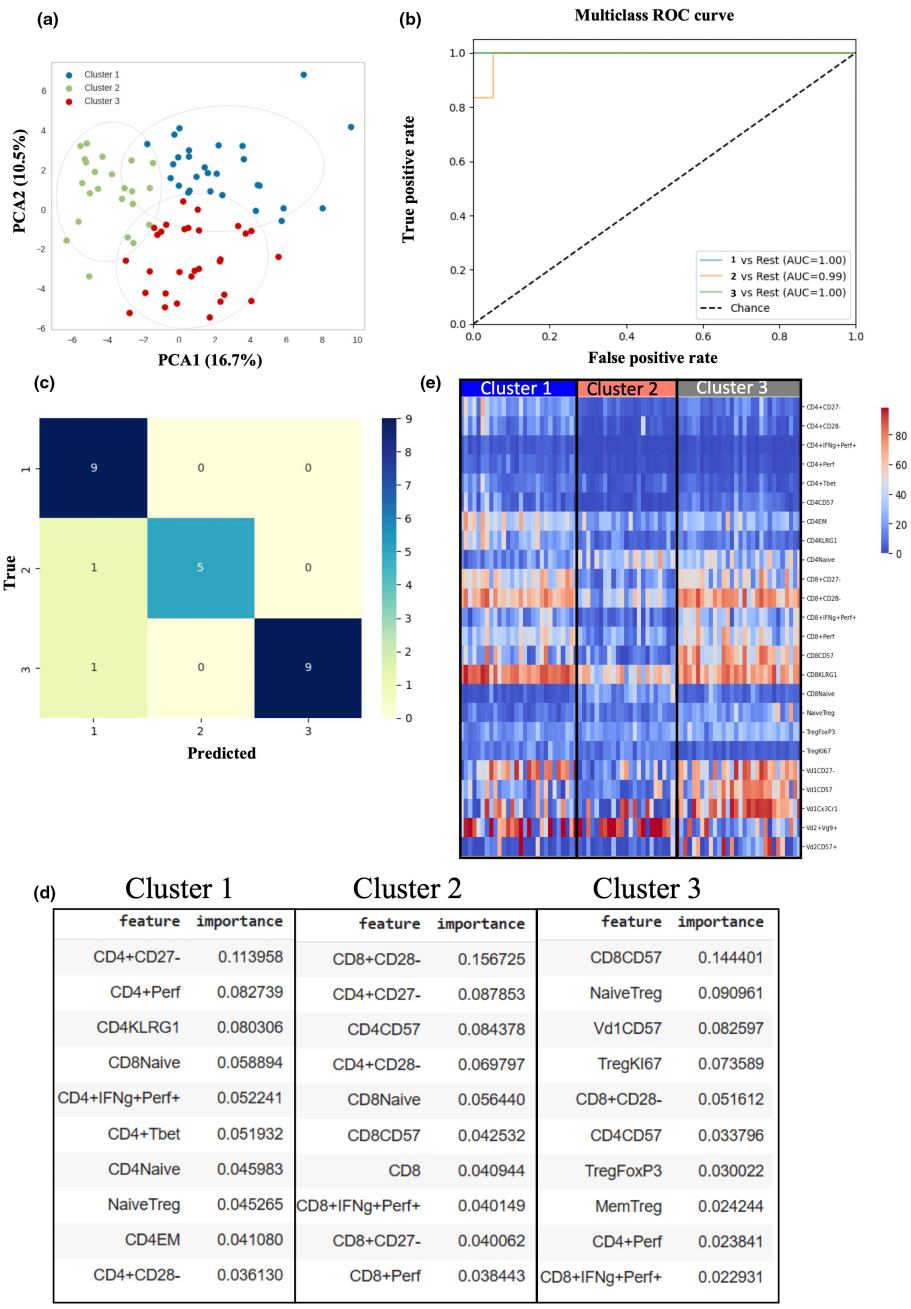
K‐means clustering of IBM patients. **(a)** Principal component analysis representing three distinct IBM clusters (cluster 1: *n* = 23, cluster 2: *n* = 28, cluster 3: *n* = 30) based on k‐means clustering. **(b)** Receiver operating characteristics (ROC) were performed using the one‐versus‐rest random forest classifier strategy. Clusters 1 and 3 lines have the same AUC value and thus appear overlaid. **(c)** confusion matrix illustrating the predicted (*x*‐axis) versus true numbers (*y*‐axis) of three IBM clusters using the random forest model. **(d)** Heatmap analysis of top features from clusters 1, 2 and 3 showing the differential expression of cell subsets between 3 IBM clusters. **(e)** List of top 10 important features calculated using mean decrease impurity method in each IBM cluster that contributed to the model predictions.

To gain greater insights into the IBM immunophenotype, we examined 66 markers in peripheral blood lymphocytes (Table 2). For CD8^+^ and CD4^+^ T cells, CD45RA and CCR7 marked the naive and memory subsets (Figure 1a). CD8^+^ T cells were mainly EM and TEMRA, with a trend towards higher TEMRA in IBM patients (IBM 40.57% vs. HC 31.78%, *P‐*value < 0.08; Table 2 and Figure 1b). CD4^+^ T cells presented predominantly naive, EM and CM subsets, yet IBM showed a larger CD4^+^TEMRA subset (IBM 3% vs. HC 1.46%, *P‐*value < 0.01, Table 2 and Figure 1c). Figure 2d highlights the most altered immune populations in IBM. The most significantly altered lymphocyte subsets in IBM that exhibited the greater increase consisted of highly activated T cells, including CD4^+^ T‐bet^+^, CD8^+^CD57^+^, CD8^+^ T‐bet^+^, CD8^+^ IFNγ^+^ Perforin^+^, CD8^+^ Perforin^+^, Natural killer T (NKT) cells, Treg Foxp3^+^ and CD8^+^CD28^−^, while Vδ2^+^, CD56^low^CD16^bright^, Vγ9^+^Vδ2^+^, Treg Ki67^+^, naïve CD8^+^ T cells and NK cells were the most reduced subsets.

**Table 2:** Proportion of peripheral blood leucocytes in IBM patients and healthy controls. All the values are represented in median (%); the values in the parentheses are range (%)

	IBM (*n* = 81)	HC (*n* = 45)	*P*‐value
CD4^+^ T cells	62.35 (29.25–96.62)	62.91 (45.58–90.21)	0.36
Naïve	26.12 (1.10–73.87)	28.08 (2.24–76.05)	0.86
Central memory	30.43 (5.90–74.79)	36.74 (13.28–74.04)	**< 0.05**
Effector memory	31.79 (8.77–76.46)	28.04 (1.14–75.28)	0.29
TEMRA	3.00 (0.20–28.63)	1.46 (0.27–15.33)	**< 0.05**
T‐bet^+^	6.04 (0.12–49.72)	1.59 (0.07–14.45)	**< 0.0001**
IFNγ^+^	12.72 (0.40–46.85)	8.89 (0.45–31.65)	0.15
Perforin^+^	2.26 (0.08–19.09)	2.31 (0.22–11.17)	0.92
IFNγ^+^ Perforin^+^	0.96 (0.00–9.99)	0.92 (0.05–9.71)	0.42
IL17A^+^	0.39 (0.04–5.16)	0.30 (0.05–2.77)	0.20
Ki67^+^	2.80 (0.42–6.77)	2.13 (0.40–6.86)	0.10
CD27^−^	11.22 (1.51–70.01)	6.29 (1.19–75.00)	0.08
CD28^−^	5.77 (0.16–58.55)	2.14 (0.06–61.84)	**< 0.01**
CD57^+^	5.28 (0.11–45.14)	3.17 (0.15–25.7)	**0.01**
KLRG1^+^	14.84 (0.91–69.5)	14 (0.67–73.2)	0.84
CD8^+^ T cells	32.59 (2.49–67.93)	28.61 (7.34–45.00)	0.08
Naïve	6.91 (0.07–53.15)	12.18 (0.25–85.71)	**< 0.05**
Central memory	4.53 (0.22–35.67)	4.41 (0.74–24.90)	0.97
Effector memory	39.77 (3.38–77.89)	37.77 (0.54–65.78)	0.80
TEMRA	40.57 (9.54–87.81)	31.78 (8.40–84.79)	0.08
T‐bet^+^	33.93 (5.28–66.46)	10.36 (0.56–43.62)	**< 0.0001**
IFNγ^+^	50.26 (1.93–92.79)	32.53 (1.75–80.74)	**< 0.001**
Perforin^+^	37.19 (2.82–77.55)	15.66 (1.47–55.39)	**< 0.0001**
IFNγ^+^ Perforin^+^	20.92 (0.04–72.94)	6.92 (0.29–26.39)	**< 0.0001**
IL17A^+^	0.28 (0.00–3.92)	0.38 (0.06–2.54)	0.23
Ki67^+^	2.79 (0.82–24.44)	2.86 (0.53–9.75)	0.71
CD27^−^	40.50 (3.50–80.63)	25.09 (3.58–66.87)	**< 0.01**
CD28^−^	63.58 (6.88–91.00)	45.98 (8.00–78.73)	**< 0.0001**
CD57^+^	36.82 (0.12–89.1)	9.72 (0.53–56.3)	**< 0.0001**
KLRG1^+^	73.13 (16.5–98)	69 (31.‐93)	0.21
Gamma delta T cells	2.15 (0.16–15.8)	3.12 (0.14–24.4)	**0.02**
Vδ2	15.40 (0–88)	46.8 (3.2–95.3)	**< 0.0001**
Vδ1	54.67 (1.4–96.3)	29.7 (0.034–90)	**< 0.001**
Vγ9^+^Vδ2^+^	47.80 (0–100)	90.50 (0–100)	**< 0.0001**
Vγ9^+^Vδ1^+^	10.40 (0–98.2)	5.26 (0–75.1)	**0.04**
Vδ2CD27^−^	18.4 (0–100)	26.3 (1.5–94.8)	0.45
Vδ2^+^ CX_3_CR1^+^	53.5 (0–100)	61.70 (1.4–96.4)	0.43
Vδ2^+^ CD57^+^	5 (0–100)	4.27 (0–98.9)	0.32
Vδ1^+^ CD27^−^	50.2 (0–96.87)	27.7 (0–96.0)	0.15
Vδ1^+^ CX_3_CR1^+^	48.8 (0–98.17)	20.5 (0–94.4)	**< 0.001**
Vδ1^+^ CD57^+^	24.5 (0–89.3)	10.1 (1.3–80.5)	**< 0.0001**
B cells	12.7 (1.1–30.6)	16.44 (5.8–31.4)	**< 0.001**
Naïve	41.1 (1.3–77.4)	35.4 (2.9–79.0)	0.66
Unswitched	2.7 (0.6–18.0)	4.4 (0.3–18.1)	**< 0.05**
Switched	8.6 (2.8–36.3)	9.6 (3.2–36.4)	0.15
Memory	43.4 (16.1–89.1)	47.7 (9.0–76.7)	0.91
Treg			
CD4^+^ CD127^−^CD25^+^	3.6 (1.1–8.0)	5.0 (2.2–10.8)	**< 0.0001**
Treg Foxp3	21.2 (6.2–45.0)	11.5 (3.6–58.9)	**< 0.0001**
Treg Ki67	8.1 (0.9–32.3)	14.3 (3.5–26.3)	**< 0.001**
Naïve	12.4 (1.1–73.0)	10.1 (2.3–72.1)	0.07
Memory	72.6 (27.3–89.5)	73.3 (26.5–90.3)	0.22
Activated	11.1 (0.1–44.8)	11.0 (1.5–31.6)	0.71
NKT in total lymphocytes			
CD3^+^CD56^+^CD16^−^	0.20 (0.02–3.9)	0.08 (0.01–1.9)	**< 0.0001**
NK cells	1.8 (0.10–8.0)	1.7 (0.2–9.8)	0.90
CD56^bright^	4.3 (0.32–50.5)	3.2 (0.2–9.3)	**< 0.05**
CD56^dim^	72.8 (6.74–95.0)	58.0 (6.0–94.4)	**< 0.05**
CD56‐CD16^bright^	13.9 (0.55–92.3)	38.6 (2.4–93.1)	**< 0.01**
Monocytes			
CD61^−^CD45^+^CD16^−^	1.9 (0.25–4.9)	1.7 (0.15–4.7)	0.20
Classical monocytes	81.3 (39.27–98.8)	82.6 (36.4–97.3)	0.72
Intermediate monocytes	13.2 (1.00–48.5)	12.6 (2.5–40.5)	0.78
Inflammatory monocytes	3.3 (0.11–26.3)	2.8 (0.05–38.1)	0.44
Classical dendritic cells			
CD3^−^CD19^−^CD14^−^CD56^−^CD16^−^CD11c^+^ HLADR^+^	0.06 (0.00–0.6)	0.05 (0.00–0.2)	0.13
Plasmacytoid dendritic cells			
CD3^−^CD19^−^CD14^−^CD56^−^CD16^−^HLADR^+^CD123^+^	0.01 (0.00–0.1)	0.01 (0.00–0.05)	**< 0.05**
Basophils			
CD3^−^CD19^−^CD14^−^CD56^−^CD16^−^HLADR^−^CD123^+^	0.12 (0.00–0.6)	0.07 (0.00–0.2)	**< 0.01**
Neutrophils			
CD45^+^CD16^+^	33.2 (1.4–64.9)	28.6 (2.8–47.9)	**< 0.05**

Naive (CD45RA^+^CCR7^+^; Naive), central memory (CD45RA^−^CCR7^+^; CM), effector memory (CD45RA^−^CCR7^−^; EM) and terminally differentiated effector memory (CD45RA^+^CCR7^−^, TEMRA).

Bold values are statistically significant *p* < 0.05.

Our phenotype datasets were assessed using a random forest (RF) classifier, achieving a 94% AUC ROC score and 86.84% accuracy (Figure 1e and f). Precision, recall, F1 score and Matthew's correlation coefficient (MCC) exceeded 70%, reflecting effective IBM discrimination (Supplementary table 1). Feature importance analysis revealed CD8^+^ T‐bet^+^, NKT, CD8^+^ IFNγ^+^Perf^+^ and more as top contributors (Figure 1g). SHAP plots showcased NKT and CD8^+^ T‐bet^+^'s positive impact at moderate‐high frequencies and negative influence at low frequencies (Figure 1h). In contrast, Vδ2^+^Vγ9^+^ and Tregs predicted IBM when reduced (Figure 1h). Overall, our analysis using a random forest classifier, feature importance and SHAP plots identified distinct immune signatures, including CD8^+^ T‐bet, NKT cells, CD8^+^ IFNγ^+^Perf^+^, CD8^+^ Perf^+^ and Vδ2^+^Vγ9^+^ in IBM patients compared to healthy donors.

### Unsupervised machine learning on peripheral immune profiles stratifies IBM patients into three distinct clusters

Next, we employed K‐means clustering (Figure 2a), an unsupervised machine learning algorithm, to analyse peripheral immune subsets; this strategy successfully stratified IBM patients into three distinct clusters: cluster 1 (*n* = 23), cluster 2 (*n* = 28) and cluster 3 (*n* = 30). To further determine the accuracy of our clustering model, we employed the random forest algorithm using the one‐versus‐rest multiclass strategy. This approach involved training multiple classifiers, one for each class, where each classifier was trained to distinguish a particular class from the rest. The resulting area under the receiver operating curve (AUC) ranged from 0.99 to 1 (Figure 2b), indicating an outstanding discrimination performance. The specificity and sensitivity of the three clusters are shown in the confusion matrix (Figure 2c) and summarised in Table 3. The sensitivity and specificity of clusters 1 and 3 were identically high at 90% and 100%, respectively, while they were slightly lower for cluster 2 at 83.3% and 95.8%. A summary of the model's metrics, including precision, recall, F1 and Matthews coefficient, is detailed in Supplementary table 2.

**Table 3:** Sensitivity and specificity of the three IBM clusters according to the confusion matrix

Cluster	Sensitivity (%)	Specificity (%)
Cluster 1	90	100
Cluster 2	83.3	95.8
Cluster 3	90	100

Subsequently, we investigated the top 10 features that contribute most significantly to the model's prediction for each of the three clusters (Figure 2d). In cluster 1, notable contributions came from CD4^+^ T‐cell populations, including CD4^+^CD27^−^, CD4^+^Perforin^+^, CD4^+^KLRG1^+^, CD4^+^IFNg^+^Perforin^+^, CD4^+^T‐bet^+^, CD4^+^EM and CD4^+^CD28^−^. These findings were further supported by a heatmap analysis (Figure 2e) and the differential analysis using ANOVA (Table 4). Notably, the heatmap analysis indicated that cluster 1 also exhibited a highly activated CD8^+^ T cell signature, characterised by an increased frequency of CD8^+^KLRG1^+^ cells and low frequency of cells expressing the co‐stimulatory molecules CD28 and CD27 compared to cluster 2. In cluster 2, the top 10 important features contributing to the model included a combination of CD8^+^ populations such as CD8^+^CD28^−^, CD8^+^CD27^−^, CD8^+^CD57^+^, CD8^+^IFNγ^+^Perforin^+^, along with CD4^+^ populations such as CD4^+^CD27^−^, CD4^+^CD57^+^ and CD4^+^CD28^−^ (Figure 2d). These features exhibited significantly lower frequencies in cluster 2 than in the other clusters. Conversely, the frequency of naïve CD8^+^ T cells was significantly higher than the other clusters (Figure 2e, Table 4). In cluster 3, the important features included markers such as CD8^+^CD57^+^, Vδ1^+^CD57^+^, CD4^+^CD57^+^ and a relatively higher proportion of CD8^+^IFNγ^+^Perforin^+^ than the other clusters. This cluster also demonstrated increased T‐reg populations such as Naïve Tregs (CD127^−^CD25^+^CD45RA^+^) and total T‐reg FoxP3^++^, indicating their contribution to the model prediction (Figure 2d and e). Differential analysis reinforced the observation of significantly increased frequency of CD57^+^ cells within T cells in cluster 3, along with elevated levels of naïve and total FoxP3^+^ Tregs (Table 4). However, there was also a noticeable decrease in proliferating Ki67^+^ Tregs compared to clusters 1 and 2.

**Table 4:** Proportion of peripheral blood leucocytes in the three IBM clusters

Marker	Cluster 1 *N* = 23	Cluster 2 *N* = 28	Cluster 3 *N* = 30	Un‐adj. *P*‐value	*P*‐adjusted		
					Cluster 1 & 2	Cluster 2 & 3	Cluster 1 & 3
Age	76 (69–98)	71 (41–84)	72 (48–89)	0.1	Ns	Ns	Ns
NOY	9 (1–21)	7 (2–28)	11 (2–20)	0.9	Ns	Ns	Ns
CD4^+^Naïve	17.5 (1.1–47.6)	38.8 (6.6–73.)	37.2 (10.3–63.4)	2.4 × 10^−6^	1.7 × 10^−5^	Ns	5.2 × 10^−5^
CD4^+^EM	38.6 (23.8–76.4)	18.0 (8.7–57.0)	27.7 (9.1–65.8)	3.9 × 10^−7^	6.5 × 10^−7^	Ns	1.7 × 10^−4^
CD4^+^CD57^+^	7.1 (0.4–45.1)	1.1 (0.1–10.7)	8.7 (0.3–28.5)	8.0 × 10^−8^	2.3 × 10^−6^	5.7 × 10^−7^	Ns
CD4^+^KLRG1^+^	25.5 (4.3–69.5)	6.5 (0.9–39.4)	9.1 (1.3–40.1)	1.4 × 10^−7^	6.8 × 10^−7^	2.0 × 10^−5^	Ns
CD4^+^CD28^−^	12.2 (2.0–58.5)	1.5 (0.3–44.0)	3.8 (0.6–26.5)	1.0 × 10^−7^	5.9 × 10^−8^	0.01	1.1 × 10^−3^
CD4^+^CD27^−^	21.7 (7.6–70.0)	4.6 (1.5–16.4)	9.6 (1.7–42.3)	2 × 10^−10^	1.2 × 10^−10^	8.4 × 10^−5^	4.8 × 10^−3^
CD4^+^T bet^+^	9.7 (3.2–49.7)	3.6 (0.1–17.8)	4.4 (0.4–18.4)	2.9 × 10^−6^	3.7 × 10^−5^	Ns	3.0 × 10^−5^
CD4^+^Perf^+^	6.2 (0.7–19.0)	1.0 (0.0–3.6)	1.3 (0.1–9.5)	1.6 × 10^−8^	9.4 × 10^−8^	Ns	5.8 × 10^−6^
CD4 + IFNγ+Perf+	3.3 (0.2–9.9)	0.4 (0.0–2.8)	0.7 (0.0–6.7)	4.3 × 10^−8^	4.1 × 10^−8^	0.04	2.0 × 10^−4^
CD8^+^Naïve	2.63 (0.07–23.0)	21.3 (3.9–53.1)	7.1 (0.7–37.3)	2.2 × 10^−9^	8 × 10^−10^	8.4 × 10^−4^	2.3 × 10^−3^
CD8^+^CD57^+^	25.2 (2.8–85.0)	10.2 (0.1–46.3)	64.7 (33.2–89.1)	4 × 10^−10^	3.1 × 10^−3^	2.0 × 10^−10^	3.9 × 10^−4^
CD8^+^KLRG1^+^	82.8 (48.8–98.0)	57.3 (16.5–78.1)	73.9 (38.5–91.1)	4.6 × 10^−8^	2.0 × 10^−8^	5.9 × 10^−4^	0.02
CD8^+^CD28^−^	65.2 (45.5–89.1)	40.1 (6.8–64.4)	76.0 (37.0–91.0)	2.4 × 10^−9^	1.5 × 10^−6^	6.1 × 10^−9^	Ns
CD8^+^CD27^−^	50.0 (22.0–78.2)	16.6 (3.5–47.1)	48.6 (12.5–80.6)	1.1 × 10^−7^	1.5 × 10^−7^	3.5 × 10^−5^	Ns
CD8^+^Perf^+^	39.2 (16.2–66.5)	20.6 (2.8–42.5)	45.5 (8.9–74.1)	1.9 × 10^−7^	1.8 × 10^−5^	4.4 × 10^−7^	Ns
CD8^+^IFNγ^+^Perf^+^	22.3 (1.4–56.3)	5.0 (0.04–46.2)	31.1 (3.0–72.9)	2.6 × 10^−7^	9.6 × 10^−4^	1.2 × 10^−7^	0.04
Naive Treg	7.4 (1.16–21.41)	11.2 (3.2–29.2)	19.3 (8.0–73.0)	4.5 × 10^−8^	Ns	7.1 × 10^−5^	9.6 × 10^−8^
TregFoxP3	15.7 (7.4–36.7)	19.8 (8.9–44.8)	30.1 (6.2–45.0)	8.7 × 10^−5^	Ns	4.3 × 10^−3^	1.1 × 10^−4^
TregKI67^+^	13.4 (3.1–32.3)	12.5 (2.8–23.1)	4.8 (1.6–11.9)	3.9 × 10^−8^	Ns	1.4 × 10^−5^	2.2 × 10^−7^
Vd1^+^CD27^−^	67.7 (0.0–96.8)	17.1 (0–73.3)	64.4 (10.5–95.2)	5.6 × 10^−5^	6.9 × 10^−4^	9.8 × 10^−5^	Ns
Vd1^+^CX3CR1^+^	31.5 (0.3–94.6)	11.7 (0–96.2)	71.8 (1.2–98.1)	1.8 × 10^−5^	Ns	7.1 × 10^−5^	4.3 × 10^−4^
Vdδ1^+^CD57^+^	23.5 (0–69.3)	10.4 (1.0–66.3)	66.2 (9.0–89.3)	3.6 × 10^−8^	Ns	5.5 × 10^−8^	1.1 × 10^−4^
Vδ2^+^Vγ9^+^	68.9 (0–100)	89.7 (0–100)	27.0 (0.0–94.3)	0.02	Ns	0.03	0.04
Vδ2^+^CD57^+^	2.6 (0–100)	1.6 (0–45.3)	20.3 (0.0–95.8)	9.9 × 10^−4^	Ns	3.5 × 10^−3^	3.8 × 10^−3^

All the values are represented in median (%); the values in the parentheses are range (%).

NOY, number of years living with IBM; Ns, not significant; Un‐adj, unadjusted.

Consequently, we interpret our three clusters as follows. Cluster 1 represents highly activated and pro‐inflammatory CD4^+^ T cells in conjunction with a differentiated CD8 profile. Cluster 2 represents a low inflammation profile and cluster 3 is characterised by the predominance of highly differentiated pro‐inflammatory CD8 and skewed gamma delta T cells.

### Impact of distinct IBM immunophenotype clusters on serological and functional features

**Figure 3:**
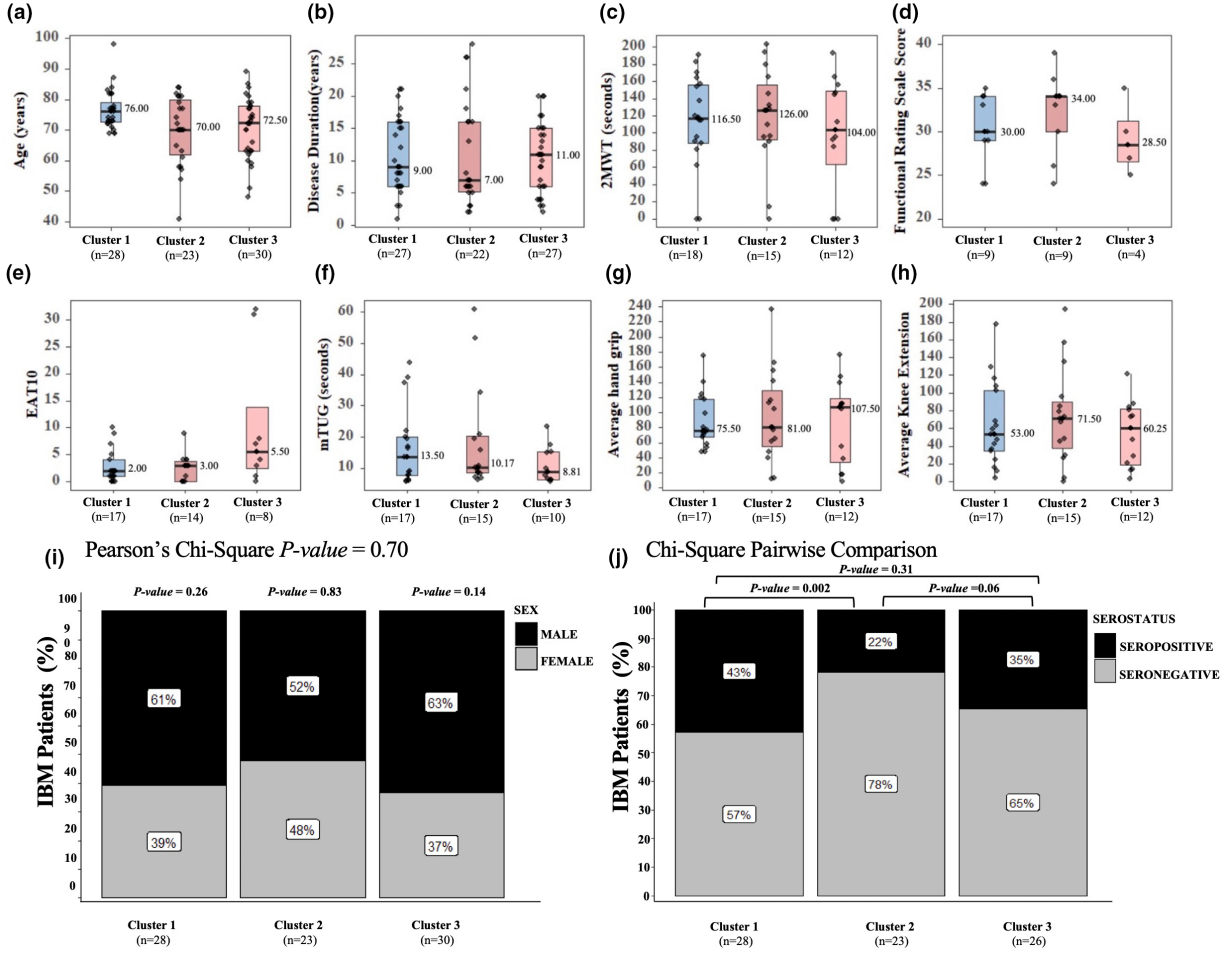
Demographic, serological and functional measures in the three defined IBM clusters. Box and Whisker plots representation of IBM patients' age **(a)** (years), number of years living with IBM **(b)** and functional measures including 2 min walk test (2MWT) in distance (metres) **(c)**, IBM Functional Rating scale (FRS) score **(d)**, EAT‐10 score **(e)**, modified Timed up and go (mTUG) score **(f)**, average left and right‐hand grip **(g)** and knee extension **(h)** measures in Newtons in clusters 1, 2 and 3. The median value for each violin plot is indicated. **(i)** Stacked bar graph showing the percentage of male to female gender distribution **(j)** and of the percentage of anti‐cN1A seropositive versus seronegative patients in the three IBM clusters. Statistical analysis of gender and serostatus ratio in each and between clusters was performed using Pearson's two‐tailed Chi‐Squared test and the Chi‐Square pairwise comparison test, respectively. The number of patients for whom measures for each of the variables were available is indicated.

To investigate the relationship of distinct immunophenotypes on the functional outcomes of IBM patients, we analysed the encompassing demographic, serological and functional parameters within the three clusters (Figure 3, Table 4). As IBM is characterised by progressive muscle deterioration, we specifically examined the age and disease duration across the three clusters to evaluate potential variations. Interestingly, we did not find significant differences for these variables between the clusters (Figure 3a and b, Table 4). Similarly, the male‐to‐female ratios exhibited no statistically significant disparities within or between the clusters (Figure 3i, Pearson's Chi‐squared *P‐*value = 0.70).

We also tested the IBM cohort for the presence of anti‐cN1A antibodies; 35% of the patients were seropositive. We further investigated the prevalence of anti‐cN1A seropositivity within the three clusters (Figure 3j). Cluster 1 demonstrated the highest proportion, accounting for 43%; conversely, cluster 2 exhibited the lowest proportion with 22%. Importantly, we observed a significant difference in serostatus between cluster 1 and cluster 2 (*P‐*value = 0.002), but not between clusters 1 and 3 or clusters 2 and 3.

To evaluate the presence of functional disparities between the IBM clusters, we utilised various clinical outcome measures, including TUG, IBM‐FRS, 2MWT, EAT10 and average quantitative muscle test scores for hand grip and knee extension strength (Figure 3c and h). Notably, we did not find evidence of significantly different functional measures between these clusters. Nevertheless, a trend could be identified for cluster 3, where patients exhibited lower scores than the two other clusters for 2MWT, and TUG yet showed the highest scores for average hand grip strength, reduced IBM‐FRS scores and increased EAT‐10 score. However, considering the clusters' low sample size, additional studies will be needed to confirm these observations.

## Discussion

Inclusion body myositis is a complex inflammatory‐degenerative disease that affects skeletal muscles, leading to progressive muscle weakness and atrophy in select muscle groups. While the exact cause of IBM is unknown, it is thought to involve a combination of autoimmune, genetic and degenerative factors.^5^ Furthermore, there is a considerable level of heterogeneity between patients, with some progressing more rapidly than others.^18^ It is currently unknown what factors are responsible for this heterogeneity and discrepancy in progression rate, but we hypothesise that the extent of variability in immunity dysregulation is a contributing factor and that immunophenotype profiling provides a potent characterisation tool that may provide insights into the disease mechanisms.

In this study, we analysed peripheral blood from IBM patients and aged controls by flow cytometry to generate snapshots of individual immunophenotypes and applied a supervised computational approach using the random forest classifier to identify immune signatures that are potentially relevant to the aetiology of IBM. In the context of inflammatory myopathies, IBM has been associated with a marked increase in CD8^+^ TEMRA cells, which are known for their resistance to apoptosis, enhanced cytotoxicity and secretion of pro‐inflammatory cytokines.^7^ Accordingly, we measured a notable abundance of CD8^+^ TEMRA cells in this IBM cohort. Importantly, our study also revealed that this lymphocyte population also predominated in healthy aged controls, suggesting that ageing‐related changes may contribute, at least in part, to this phenomenon. Interestingly, we found no correlation between the frequency of CD8^+^ TEMRA cells and age in either the IBM or control group (Supplementary figure 1a, b), suggesting that other factors, such as infection history, might influence their accumulation.^28^ To further explore the potency of the CD8^+^ TEMRA subset variability in discriminating between IBM and healthy individuals, we employed a random forest model. These cells did not emerge as a top‐ranking feature in the model, which suggested their limited contribution to the model's discriminatory power between IBM and HC. This finding raised intriguing questions about the true impact of CD8^+^ TEMRA cells in the immunological landscape of IBM and prompted us to explore alternative factors that possibly contribute to the disease pathology.

Moreover, we found that in the IBM group, CD8^+^ T cells predominantly exhibited a loss of the co‐stimulatory receptors CD27 and CD28, which aligns with previous findings.^6^, ^29^ Notably, these changes were more pronounced in the CD8^+^ T‐cell population, although significant alterations in CD4^+^ and gamma‐delta T cells were also detected. Specifically, IBM patients possessed an increased proportion of CD4^+^ effector memory cells with an inflammatory Th1 T‐bet^+^ profile and displaying a late‐differentiated phenotype characterised by CD57 upregulation and loss of CD28. These findings demonstrate that both the CD8^+^ and CD4^+^ compartments were dysregulated, which likely contributes to the immunopathology associated with IBM.

Furthermore, we observed intriguing changes in the gamma‐delta T‐cell population. The IBM patients exhibited an altered ratio of Vδ2^+^ to Vδ1^+^ cells, along with a significantly reduced Vγ9^+^Vδ2^+^ subset, which typically dominates the peripheral gamma‐delta T‐cell pool in healthy individuals. Additionally, the Vδ1^+^ subset showed increased expression of CD57 and CX3CR1, indicating a skewed profile towards a highly differentiated phenotype. The semi‐invariant Vγ9^+^Vδ2^+^ cells possess innate‐like features that strongly diverge from the Vγ9^−^Vδ2^+^ and Vδ1^+^ phenotype; indeed, these two subsets have been found to undergo clonal expansion and differentiation, like adaptive cells, following acute infection.^30^, ^31^ More generally, the Vδ1^+^ cells have been found to dominate following cytomegalovirus^32^ and Epstein–Barr (EBV)^33^ virus infections. Likewise, it is possible that the sustained inflammatory conditions in IBM drive the changes observed within the gamma‐delta T‐cell population.

The random forest classifier model identified CD8^+^ T‐bet^+^ as a prominent feature in IBM. T‐bet, a transcription factor expressed in various innate and adaptive immune cells, plays a critical role in regulating immune cell differentiation and function, notably in promoting pro‐inflammatory cytokine production and cytotoxic T cell differentiation. Consistent with our findings, Dzangué‐Tchoupou and co‐workers also reported CD8^+^ T‐bet^+^ cells as a potential biomarker for IBM using different ML approaches from ours. Their study demonstrated that a proportion of CD8^+^ T‐bet^+^ cells > 51.5% had high accuracy for distinguishing IBM from other types of myositis (sensitivity of 94.4%, specificity of 88.5% and an area under the curve of 0.97).^21^ Our independent validation strengthens CD8^+^ T‐bet^+^ as a potential IBM biomarker.

In this study, we have applied predictive modelling to identify immune changes in IBM compared to HC's samples, such as an increase in CD8^+^ T‐bet, in order to unveil significant insights into the intricate processes at play and gain a deeper understanding of the disease's mechanistic pathways. We also identified a moderate positive correlation between CD8^+^ T‐bet^+^ and CD8^+^ TEMRA cells (Spearman's *P‐*value = 0.40, Supplementary figure 3). However, as a result of the technical limitations of the flow cytometer used in this study, we were unable to combine the TEMRA and T‐bet markers into the same antibody panel for CD8^+^ cell analysis and therefore could not confirm that TEMRA cells were also T‐bet^+^. Nevertheless, the abundance of CD8^+^ T‐bet^+^ that we detected in IBM, its identification as a top feature in the random forest model and the positive correlation with CD8^+^ TEMRA cells, together suggest that the CD8^+^ TEMRA population is likely to be predominantly T‐bet^+^.

We also performed an unsupervised cluster analysis to stratify IBM patients based on distinct immunophenotypes. Impressively, despite the data available being limited to 81 patients, our model successfully identified three distinct clusters. Cluster 1 displayed a distinctive CD8^+^ T‐cell profile characterised by a high degree of differentiation. Additionally, the top contributing features for cluster stratification primarily comprised various CD4^+^ T‐cell populations, including CD27^−^, KLRG1^+^, T‐bet^+^ and perforin^+^, suggesting the presence of a profoundly differentiated cytotoxic profile. The prevalence of anti‐cN1A seropositivity was the highest in this cluster and was significantly increased compared to cluster 2; this result prompts further investigation into the direction of the causal relationship between cell‐mediated inflammatory and cytotoxic conditions and anti‐cN1A production. Cluster 2 included patients exhibiting a distinct immunological profile characterised by reduced inflammation markers, as evidenced by the substantial decrease in all markers listed in the feature importance plot compared to clusters 1 and 3. Notably, this cluster displayed higher counts of CD8^+^ and CD4^+^ naïve T cells. Additionally, we did not observe an altered gamma delta T cell subset distribution in this cluster. These findings underscore the need for further studies to elucidate the role of gamma‐delta T cells in IBM.

Recently, the presence of CD8^+^ large granular lymphocytes (LGLs) has been revealed in the blood and muscle of approximately 34–58% of IBM patients.^34^, ^35^ In line with these findings, cluster 3 further substantiates the importance of these late‐differentiated T cells in IBM. Notably, patients in this cluster also possess an abundance of CD4^+^ and gamma‐delta (Vδ1 and Vδ2) T cells exhibiting high expression levels of CD57. Interestingly, even though it has been reported that circulating regulatory T cells are found at a reduced frequency in IBM,^10^ cluster 3 exhibits the highest proportion of total FoxP3^+^ and naïve Tregs of all clusters, suggesting the presence of regulatory mechanisms aimed at counteracting the pathological impact stemming from highly differentiated and inflammatory T cells. However, the apparent absence of proliferating Tregs poses a challenge to this interpretation. Alternatively, studies focusing on the role of Treg cells in autoimmune and inflammatory disorders have unveiled a phenomenon wherein these cells acquire T helper‐like phenotypes and heightened expression of pro‐inflammatory cytokines while still retaining Foxp3 expression.^36^, ^37^, ^38^ Therefore, it cannot be excluded that the identified Treg population might potentially contribute to the notably dysregulated T‐cell profile in cluster 3.

It is worth noting a trend of increased disease severity in cluster 3's patients compared to the other two clusters. This trend is supported by lower scores on functional measures such as the mTUG and 2MWT that reflect a reduction of leg muscle strength, while in contrast stronger average hand grip values were measured. The patients in this cluster have reported a more reduced ability to perform daily tasks resulting in lower IBM‐FRS values than the other clusters' patients. A higher level of dysphagia was also suggested by the higher average EAT‐10 score measured, including some patients with very high scores that translate as a much‐impaired swallowing function. We also note that cluster 3 has a longer disease duration with a median value of 11 years. However, the data distribution of this variable is normal in this cluster, with a large part of the measures that overlap most of those in the other 2 clusters. This suggests that the more pronounced disease severity measures reported in cluster 3 are not reflecting the sole effect of longer disease duration. Whether the immune changes that we reported here are directly or indirectly responsible for the modulation of disease severity should be the scope of future studies that will delve into the particular immunopathogenic mechanisms of IBM.

This study has limitations that should be taken into consideration while interpreting the findings. Firstly, it is a retrospective analysis conducted at a single centre, which limited the number of participants in both the IBM and HC cohorts and restricted the generalisation of the results. Another limitation stems from the incomplete dataset of recorded functional outcome measures; as a result of these constraints, only a limited proportion of patients in the clusters could be assessed. Also, the stratification of patients into 3 clusters further decreased the sample size of each of these subgroups. Although, our data suggest that the immunophenotype associated with cluster 3 is associated with increased disease severity, future studies involving larger patient cohorts will be required to confirm these preliminary findings. This underscores the need for future studies to include comprehensive prospective functional outcome assessments. Finally, while machine learning models were employed in this study, they were not externally validated. To mitigate the risk of overfitting, the dataset was divided into training and validation sets using the train‐test‐split function with the stratify parameter. This approach ensured that the distribution of target classes in the training and testing datasets was balanced and representative of the original dataset. However, future studies with completely independent test data that are not used during model development will be required in order to evaluate the performance and generalisability of the ML models accurately. In addition, longitudinal studies monitoring the immune and functional changes of stratified IBM patients may provide valuable insights into how the disease progresses and offer improved patient management strategies.

## Conclusion

Through phenotypic analyses of peripheral blood leucocytes and advanced computational modelling, our study made substantial strides in unravelling the immunological shifts linked to IBM. Our findings not only reaffirm previous insights into aberrant T cell alterations, notably heightened CD8^+^ T‐bet^+^, but also achieve refined stratification of IBM patients via distinct immunophenotypic profiles. However, the clinical and functional ramifications of these immune phenotypes remain elusive. This investigation forms a robust foundation for delving deeper into the functional significance of CD8^+^T‐bet^+^ and CD8^+^CD57^+^, alongside discrete immune subsets such as γδ T cells and regulatory T cells. These findings provide a strong rational for future studies using the same approach to compare IBM cohort to cohorts affected by other inflammatory myopathies and to identify specific IBM biomarkers that may distinguish the disease from other IIMs. Comprehending these implications holds potential for future clinical applications, spanning IBM diagnosis, prognosis and management.

## Methods

### Study population

**Figure 4:**
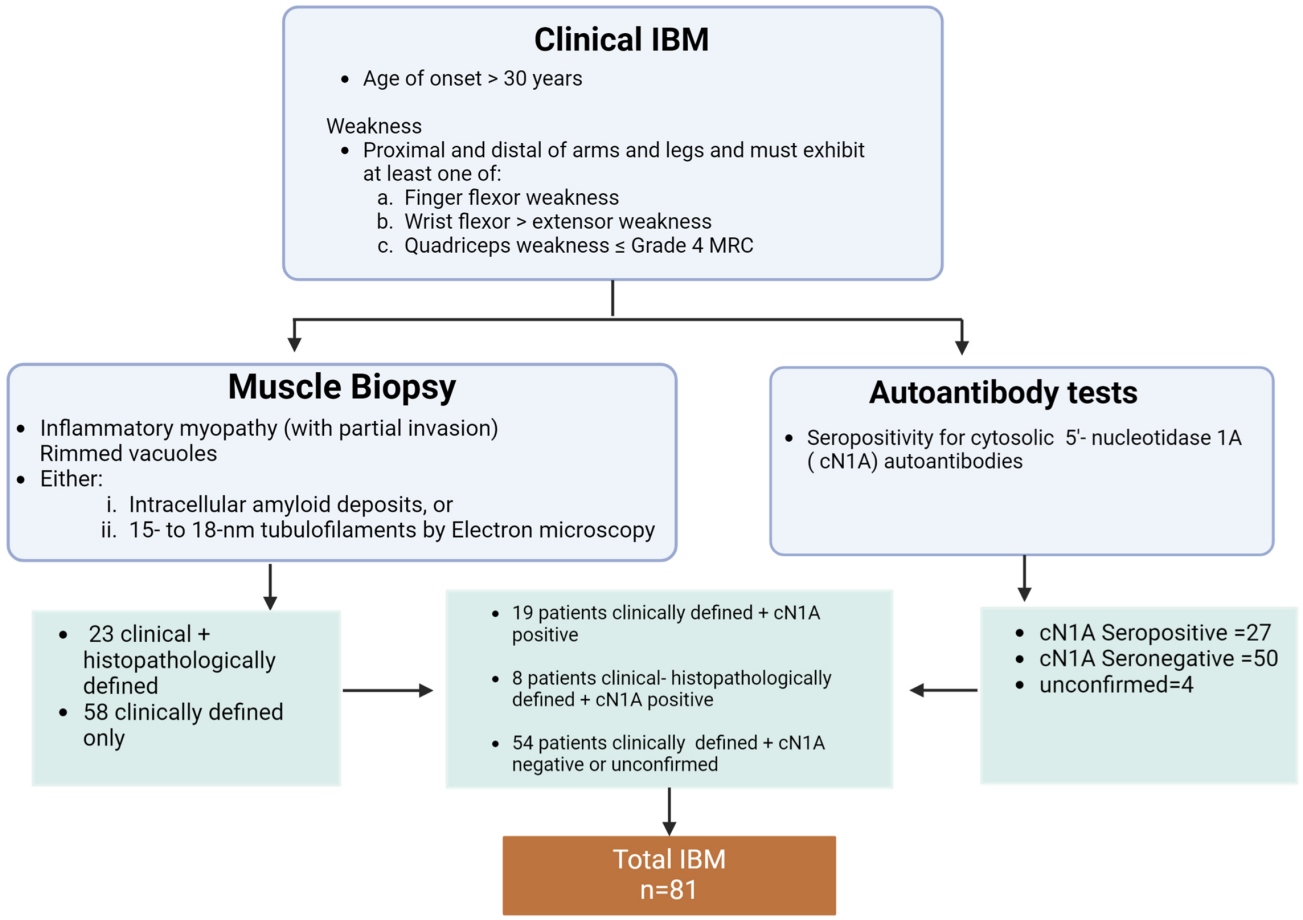
Schematic of IBM inclusion criteria. Criteria include a combination of clinical, histopathological and laboratory findings (serology for autoantibodies against cN1A). Our total cohort consisted of 81 IBM patients. This figure was created using BioRender.com.

A total of 81 patients diagnosed with IBM, by a consultant neurologist were enrolled in this study. Recruitment occurred between 2017 and 2022 from specialist myositis clinics at Murdoch University and the Perron Institute in Perth, Western Australia, for inclusion criteria and patient stratification, see Figure 4. Additionally, 45 age‐matched healthy individuals without a muscle, autoimmune or chronic inflammatory disease and naïve to any immune‐modulating drugs were recruited as controls. Blood samples were collected into lithium heparin vacutainer tubes (Becton Dickinson Bioscience, VIC, Australia) and processed within 2 h of being collected. Written informed consent was obtained from all participants prior to the collection of blood. Samples and clinical data were processed and analysed in a de‐identified manner. Ethical approval for the study was obtained from the Murdoch University Human Research Ethics Committee (2015/111 and 2020/188).

### Immunophenotyping of peripheral blood immune cells

Whole blood was stained with six panels of fluorochrome‐conjugated antibodies as listed in Supplementary table 3, as follows: the incubation with antibody mixes for 30 min in the dark at room temperature (RT) was followed by red cell lysis using 2 mL of FACS lysing solution (Becton Dickinson Bioscience) for 10 min at RT; samples were then washed twice in PBS (Gibco Thermo Fisher Scientific, Perth, WA, Australia) and resuspended in PBS with 2% foetal calf serum (Fisher Biotech, Wembley, WA, Australia). For cell count normalisation, counting beads (Beckman Coulter, Sydney, NSW, Australia) were added prior to data acquisition on a flow cytometer.

For intracellular cytokine analysis, blood lymphocytes were initially stimulated *in vitro* with 100 ng mL^−1^ phorbol 12‐myristate 13‐acetate (PMA; Sigma‐Aldrich, Castle‐Hill, NSW, Australia) and 1 μg mL^−1^ ionomycin (Sigma‐Aldrich) in the presence of 2 μg mL^−1^ of monensin (Sigma‐Aldrich) for 4 h at 37°C in 5% CO_2_ atmosphere. Surface staining was performed as described above and staining of the intracellular IFNγ, Perforin and IL17A content was performed after fixation and permeabilisation using the Cytofix/Cytoperm Fixation/Permeabilisation Kit (BD Bioscience) following the manufacturer's recommendations. Staining for nuclear transcription factors FoxP3 and T‐bet, and Ki‐67 protein was performed using the Transcription Factor Buffer Set (BD Bioscience) following the manufacturer's instructions.

To ensure complete data for the machine learning models, flow cytometry analysis of fresh blood samples with missing values in the data was repeated using the matching cryopreserved peripheral blood mononuclear cells (PBMCs) samples. Cells were thawed in a 37°C water bath, subsequently, gently dispensed as single drops into a 15 mL tube containing 10 mL of PBS with 5% FCS and underwent two wash cycles before being resuspended in PBS at a final concentration of 1 × 10^6^ cells mL^−1^. Surface staining was conducted by adding 50 μL antibody cocktail mix to 200 μL of PBMCs and incubating for 20 min at ambient temperature. After two additional washing steps in PBS with 5% FCS, 2.5 μL of 7AAD was added and left for 15 min prior to acquisition on a flow cytometer. For panels designated for intracellular staining, PBMCs were incubated with a 1:1000 dilution of either FVS520 (Panel 3) or FVS510 (Panel 4) for 10 min, followed by washing. Subsequently, the cells underwent both surface and intracellular staining procedures as described above for whole blood samples.

All the samples were analysed using a Beckman Coulter Gallios Flow cytometer. Data were analysed using Beckman Coulter Kaluza™ v.2.2 for Windows (Beckman Coulter, Indianapolis, IN, USA) and Flowjo™ v.10.5.3 for Windows (Flowjo™ Software Inc., Ashland, OR, USA). The gating strategies used for analysis are summarised in Supplementary figures 4–9.

### Anti‐cN1A ELISA

Anti‐cN1A antibodies were assessed using a semi‐quantitative ELISA protocol as outlined in McLeish et al.^35^ and Bundell et al.^39^ Briefly, 10 μg mL^−1^ of cN1A protein (GenScript, NJ, SA) were coated onto 96‐well flat‐bottom plates in 50 mM carbonate–bicarbonate buffer pH 9.6 for 2 h at RT. Plates were blocked overnight with 5% skim milk powder in PBS Tween at 4°C. Patient serum (diluted in blocking buffer) was added for antibody capture, incubating for 2 at RT. After washing, HRP‐conjugated anti‐human secondary antibodies (Invitrogen, Rockford, IL, USA) were added for 1 h at RT. Following washes, TMB solution (Thermo Fisher Scientific, Waltham, MA, USA) was used for revelation and 2 M H_2_SO_4_ was used as a stop solution. Absorbance at 450 nm was read using a microplate reader. Positive control was a seropositive patient's serum and pooled healthy controls' sera acted as a negative control. Blank absorbance values were subtracted from control and test sample values. Test sample measures were recorded as fold change relative to averaged negative control values. Positivity cut‐off aligned with the 99th percentile of healthy serum pool samples.^39^

### Clinical outcome measures

Clinical outcome measures were assessed by the attending neurologist and physiotherapist. To avoid inter‐rater variability, a single assessor was responsible for evaluating and scoring the patients Clinical measures used included: the 2‐min walk test, (2MWT),^40^ modified timed‐up and‐go test measures (mTUG),^41^ Eating assessment tool‐10 (EAT‐10),^42^, ^43^ IBM Functional rating scale (IBM‐FRS)^44^ and quantitative muscle testing (QMT). For QMT, we analysed quadriceps strength (knee extension) and forearm strength (hand grip). Tests were performed using a Citec Hand‐Held Dynamometer (Rijksstraatweg, CR Haren, The Netherlands). The in‐depth protocol for how each muscle group assessed has been outlined in.^45^ Tests were performed two times on each limb for grip strength and three times on each limb for quadricep strength with a 30‐s rest between each measurement; the final score represents the average measures in Newtons.

### Supervised machine learning analysis

#### Classification algorithms

To distinguish between disease phenotypes based on discrete subsets of immune cells, we employed supervised machine‐learning methods. To determine the best model for the data, we tested a logistic regression and three ensemble learning algorithms – random forest, Gradient Boosting and XGBoost (see Supplementary figure 10 and Supplementary table 4). The dataset was randomly divided into training (70%) and testing (30%) sets using the train_test_split function from Scikit‐learn.^46^ The stratify parameter was used to balance the distribution of target classes in the training and testing datasets, ensuring that the proportion of target classes in the training and test datasets was similar to the proportion in the original dataset. The model was validated in the remaining samples and the performance was evaluated using multiple metrics such as MCC, receiver operating characteristic (ROC), precision, recall and F1‐score. For the full code, see Github repository https://github.com/Emilyjane994/Immunophenotyping-in-IBM (AllModels.ipynb).

#### Random forest model

**Figure 5:**
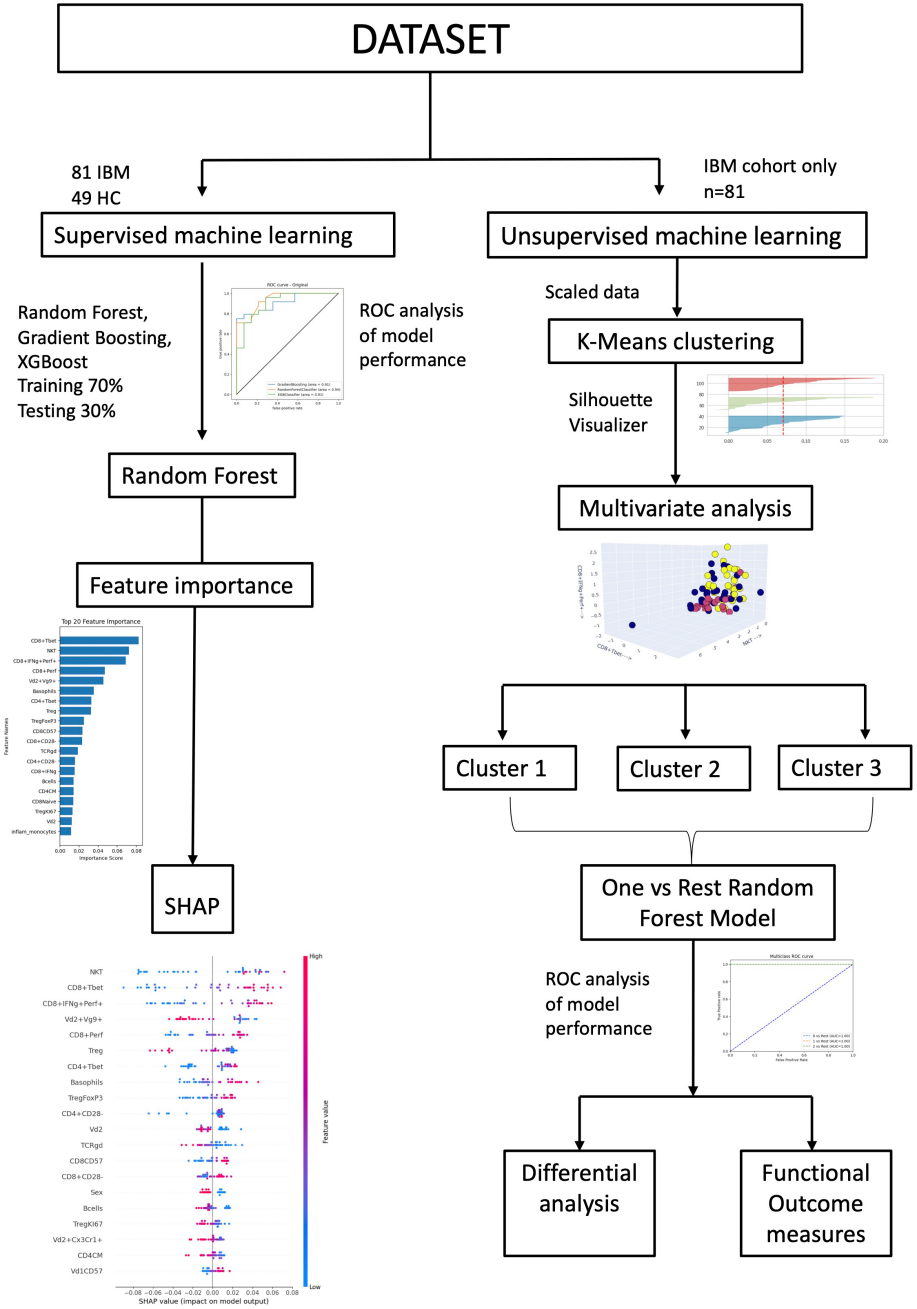
Overview of machine learning methodological pipeline. Supervised machine learning methods including random forest, Gradient Boosting and XGBoost were applied to the IBM (*n* = 81 patients) and aged‐matched healthy control (*n* = 49) cohorts to classify disease phenotypes based on immune cell subsets. Random forest was identified as the best method based on evaluation metrics. Feature importance analysis was performed using SHAP plots. Unsupervised machine learning was applied to IBM patient samples (*n* = 81) using K‐Means clustering after scaling the data. The optimal number of clusters (3) was determined using silhouette visualiser.

We employed five‐fold cross‐validation by shuffling the data and splitting it into folds, with a random seed set for reproducibility using the ‘StratifiedKFold’ function (n_splits = 5, shuffle = True, random_state = 4).^46^ The random forest model consisted of 100 decision trees (n_estimators = 100) and the random seed was set to zero (random_state = 0) for reproducibility purposes after parameter tuning. We used the mean decrease impurity method to calculate feature importance, while SHapley Additive exPlanations (SHAP) plots provide a visual representation of how each feature contributes to the model predictions, offering insights into their impact and interaction. Figure 5 details an overview of our machine learning pipeline. For the full code see GitHub repository https://github.com/Emilyjane994/Immunophenotyping-in-IBM (RandomForest.ipynb).

### Unsupervised machine learning analysis

#### K‐means clustering

K‐means clustering is an unsupervised method for grouping objects based on their similarity. We standardised the data of immune subsets in IBM patients and computed the distance of the correlation matrix in Euclidean distance. We then used the scikit‐learn library in *Python* to implement the k‐means clustering method.^46^ We used the K means function with three clusters and specified the k‐means++ initialisation method for the centroids of the clusters. The algorithm was run ten times with a maximum of 100 iterations using KMeans (n_clusters = 3, init = ‘k‐means++’, n_init = 10, max_iter = 100, random_state = 0). The optimal number of clusters was determined by the silhouette visualiser instance with K means instance. The silhouette score measures the quality of clustering by assessing how well each sample fits within its assigned cluster compared to other clusters (Supplementary figure 11). Each plot represents a different number of clusters, ranging from 2 to 5. The *x*‐axis indicates the number of clusters (K), while the *y*‐axis represents the silhouette scores. The dotted line in each plot indicates the average silhouette score across all samples. The silhouette scores range from −1 to 1, with higher scores indicating better clustering results. Values close to 1 indicate that samples are well‐clustered and clearly separated, while values close to 0 suggest overlapping or ambiguous clusters. Negative scores imply that samples may have been assigned to incorrect clusters.

#### Feature importance in each cluster

To identify the important features of each cluster, we employed the random forest algorithm, a supervised learning approach. We used the mean decrease impurity method^47^ to calculate the feature importance and selected the top 10 features for each cluster. A heatmap was created to visualise the results and identify the critical features that distinguish each cluster, potentially gaining insights into the underlying biology of the immune subsets in IBM patients. Figure 1 details an overview of our machine learning pipeline. For the full code, see GitHub repository https://github.com/Emilyjane994/Immunophenotyping-in-IBM (IBM clusters.ipynb).

### Statistical analyses

Flow cytometry data were analysed using Beckman Coulter Kaluza™ v.2.2 for Windows and Flowjo™ v.10.5.3 for Windows Statistical analyses were performed using both RStudio™ (version RStudio 2022.12.0, Integrated Development for R. RStudio, PBC, Boston, MA, USA)^48^ and Python (Python Software Foundation. Python Language Reference, version 3.10.12. URL: https://www.python.org) the latter executed within the Google Collaboratory platform. Each data set was assessed for normality using the Shapiro–Wilk normality test. A Mann–Whitney *U‐*test was used for non‐parametric data to compare the patient and healthy control groups. To evaluate the differences between IBM cluster groups, we first assessed the normality of the data distributions using the Shapiro–Wilk test (Supplementary table 5). A *P*‐value < 0.05 rejects the null hypothesis, implying that the data are not normally distributed. The non‐normally distributed populations were submitted to the Kruskal–Wallis test, followed by Dunn's post‐hoc test with Holm's correction to adjust for multiple comparisons. Populations demonstrating normality, based on the Shapiro–Wilk test, were tested with Levene's test to verify homoscedasticity to assess whether variances were equal across groups. *P*‐values > 0.05 indicate homogeneity of variances, allowing an Analysis of Variance (ANOVA) test followed by Tukey's Honest Significant Difference (HSD) post‐hoc test for multiple comparisons. To determine the influence of biological sex on the dependent variables (immune cell populations) and the pathological status group (IBM and HC), we stratified both the IBM and HC groups into male and female subgroups and performed a Kruskal–Wallis ANOVA test and Dunn's *post hoc* comparisons test on significant populations (Supplementary figure 12 and Supplementary table 6). To determine the influence of age on the dependent variables (immune cell populations), we set out to perform an analysis of covariance (ANCOVA). First, we tested the assumptions necessary for ANCOVA, including removing extreme outliers using *Z*‐scores > 2, testing for linearity using a linear regression analysis, testing for homoscedasticity using Levene's test and assessing normality of residuals using the Shapiro–Wilk test. All populations failed testing for linearity (Supplementary figure 13 and Supplementary table 7) and transformations using np.log1p function (log1p(*x*) = log(1 + *x*)) did not resolve the issue. Thus, we conducted a Spearman's rank analysis for non‐parametric data to examine the correlations between each cell population and age (Supplementary figure 1a, b). The Fisher's exact test, Pearson's Chi‐squared test and the Chi‐square pairwise comparison were used for categorical data. For ML model comparison, we used the area under the receiver operating characteristics (AUROC) using the DeLong method and MCC. The MCC is a useful metric for evaluating binary classification, especially for imbalanced datasets. AUROC is a performance metric that provides a summary of the diagnostic ability of a binary classifier system. The AUROC estimates the overall trade‐off between the true‐positive rate (sensitivity) and the false‐positive rate (1−specificity) at various discrimination thresholds. A high AUROC (> 70%) was considered good. A two‐sided *P‐*value < 0.05 was considered statistically significant. The number of asterisks indicates the level of significance of *P‐*values: *< 0.05, **< 0.01, ***< 0.001 and ****< 0.0001. For the full code, see: https://github.com/Emilyjane994/Immunophenotyping-in-IBM (Data Explanation and stats.ipynb).

## Author contributions

**Emily McLeish:** Conceptualization; data curation; formal analysis; investigation; methodology; writing – original draft. **Anuradha Sooda:** Conceptualization; data curation; formal analysis; investigation; methodology; writing – review and editing. **Nataliya Slater:** Data curation; formal analysis; investigation; methodology; writing – review and editing. **Kelly Beer:** Project administration; resources; writing – review and editing. **Ian Cooper:** Investigation; methodology; resources; writing – review and editing. **Frank L Mastaglia:** Conceptualization; writing – review and editing. **Merrilee Needham:** Conceptualization; resources; supervision; validation; writing – review and editing. **Jerome D Coudert:** Conceptualization; formal analysis; funding acquisition; investigation; methodology; project administration; supervision; validation; writing – review and editing.

## Conflict of interest

The authors declare no conflict of interest.

## Declaration of generative AI and AI‐assisted technologies in the writing process

During the preparation of this work, ChatGPT 3.5 was utilised for assistance with punctuation and reducing content during the revision process. After using this tool the authors reviewed and edited the content as needed and take full responsibility for the content of the publication.

## Supporting information


Supplementary figure 1
Supplementary figure 2
Supplementary figure 3
Supplementary figure 4
Supplementary figure 5
Supplementary figure 6
Supplementary figure 7
Supplementary figure 8
Supplementary figure 9
Supplementary figure 10
Supplementary figure 11
Supplementary figure 12
Supplementary figure 13
Supplementary table 1
Supplementary table 2
Supplementary table 3
Supplementary table 4
Supplementary table 5
Supplementary table 6
Supplementary table 7

## Data Availability

The data that support the findings of this study are openly available in our GitHub respository (Emilyjane994/Immunophenotyping‐in‐IBM) at https://github.com/Emilyjane994/Immunophenotyping‐in‐IBM Reference number (10.5281/zenodo.10605850).
