# Supplementary material for: Identification of distinct immune signatures in inclusion body myositis by peripheral blood immunophenotyping using machine learning models
Source: Clin Transl Immunology. 2024 Apr 3;13(4):e1504. doi: 10.1002/cti2.1504 (PMC10990804; doi:10.1002/cti2.1504)
Supplement: Supplementary file 1 — Supplementary figure 1 Supplementary figure 2 Supplementary figure 3 Supplementary figure 4 Supplementary figure 5 Supplementary figure 6 Supplementary figure 7 Supplementary figure 8 Supplementary figure 9 Supplementary figure 10 Supplementary figure 11 Supplementary figure 12 Supplementary figure 13 Supplementary table 1 Supplementary table 2 Supplementary table 3 Supplementary table 4 Supplementary table 5 Supplementary table 6 Supplementary table 7 [file CTI2-13-e1504-s001.pdf]

Supplementary Material

Identification of relevant biomarkers for Inclusion Body Myositis by peripheral blood immunophenotyping using machine learning models.

Emily McLeish[1*], Anuradha Sooda[1*], Nataliya Slater[1], Kelly Beer[1,2], Ian Cooper[1,2], Frank L Mastaglia[2], Merrilee Needham[1, 2,3,4], Jerome D Coudert[1,2,3]

[1] Murdoch University, Centre for Molecular Medicine and Innovative Therapeutics, Murdoch, Western Australia (WA), Australia.

[2]Perron Institute for Neurological and Translational Science, Nedlands, WA, Australia.

[3]University of Notre Dame Australia, School of Medicine, Fremantle, WA, Australia.

[4] Fiona Stanley Hospital, Department of Neurology, Murdoch, WA, Australia.

[*] These authors equally contributed to this work

This page is intentionally left blank.

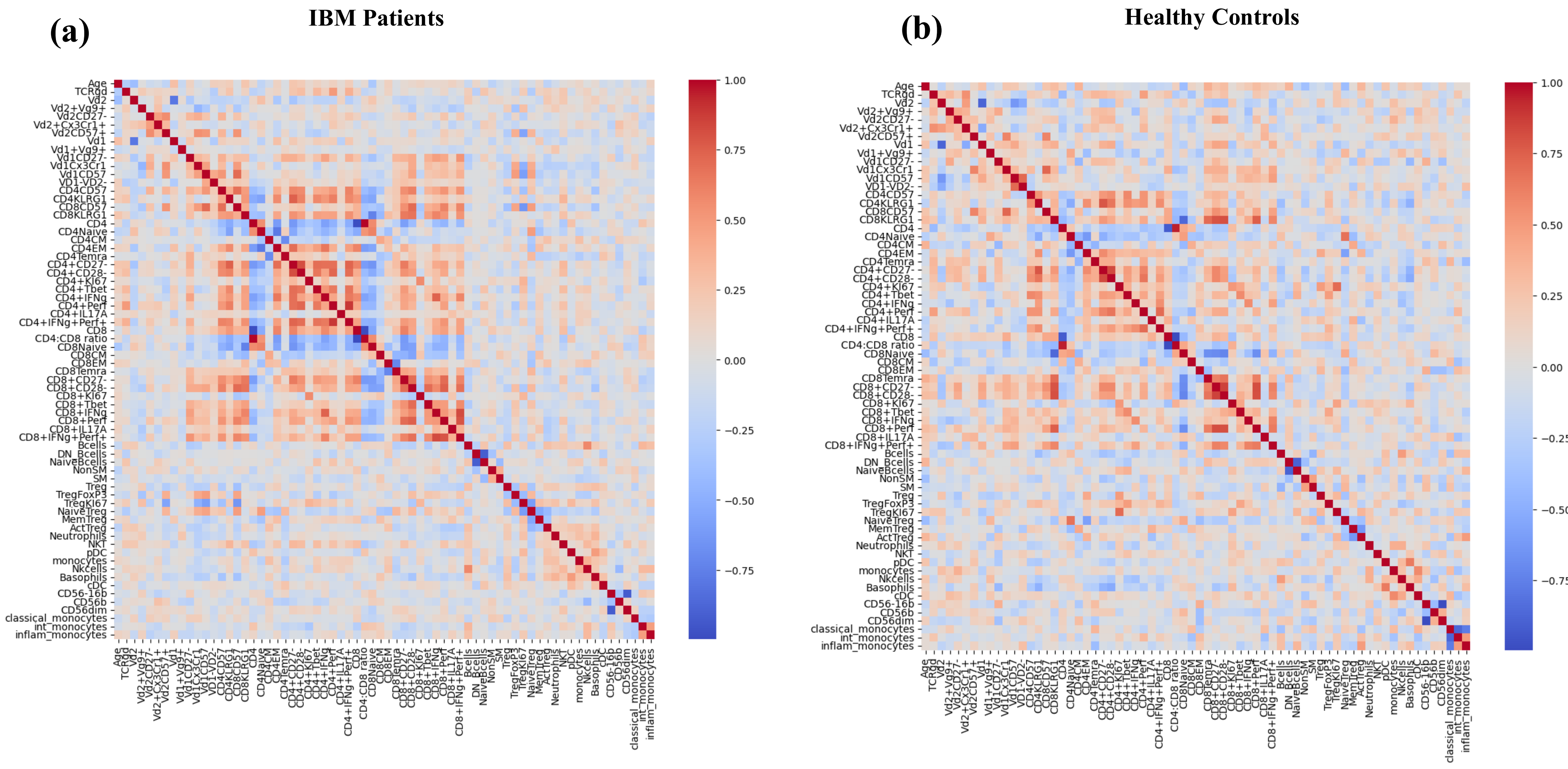

Supplementary figure 1: Correlation matrix between peripheral immune cell subsets in **(a)** IBM patients n=81 and **(b)** healthy controls n=45). Correlation analysis was performed using Spearman's rank correlation coefficient.

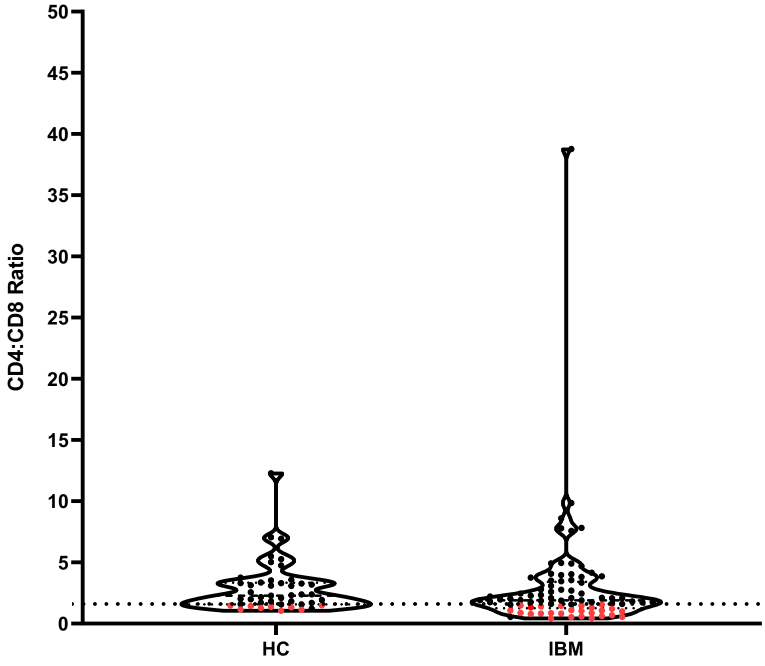

Supplementary figure 2: Violin plot showing the CD4:CD8 ratio in IBM (n=81) and HC (n=45) and the number of individuals with ratios less than 1.5 (red dots and horizontal dotted line). Statistical analysis was performed using the Mann-Whitney *U*-test for non-parametric data.

Supplementary table 1: Performance comparison of the Random-Forest model between IBM and HC.

Model	Matthews Coefficient	ROC_AUC score	Precision score	Recall score	F1 score
**Random Forrest**	0.73	0.94	0.83	0.1	0.91

Supplementary table 2: Performance comparison of the Random-Forest model with the multi-class classification of three different IBM clusters.

Model	Matthews Coefficient	Precision score	Recall score	F1 score
**Random Forrest**	0.91	0.93	0.92	0.92

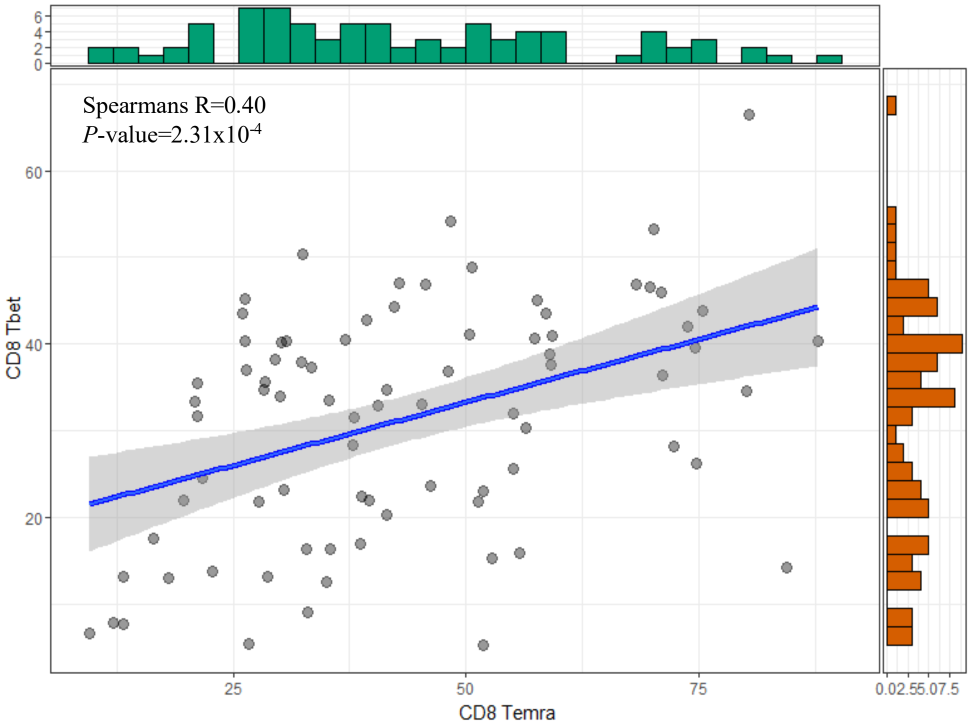

Supplementary figure 3: Spearman's Correlation analysis between the proportion of CD8+ TEMRA and $CD8^+$ T-bet$^+$ cells in IBM patients (n=81). Statistical analysis was performed using Spearman rank correlation coefficient analysis. Bar graphs represent the frequency distribution of X variables (green bar graphs) and Y variables (orange bar graphs).

Supplementary table 3. Antibodies used for immunophenotyping of peripheral blood.

Antibody	Clone	Fluorochrome	Supplier
**CD197 (CCR7)**	3D12	PE	BD
**CD4**	OKT4	FITC	BioLegend
**IgD**	IA6-2	FITC	BD
**CD56**	B159	PE CF594	BD
**TCRab**	IP26	PE Cy7	BioLegend
**CD45RA**	HI100	APC	BioLegend
**CD8**	SK1	APC H7	BD
**CD19**	HIB19	APC FIRE750	BioLegend
**CD27**	M-T271	BV421	BD
**CD3**	UCHT1	BV510	BD
**CD127**	HIL-7R-M21	BB515	BD
**CD127**	HIL-7R-M21	APC R700	BD
**CD25**	M-A251	PE	BD
**CD28**	CD28.2	PE Cy7	BD
**CD4**	OKT4	AF 700	BioLegend
**CD19**	HIB19	FITC	BD
**CD61**	VI-PL2	FITC	BD
**CD123**	6H6	PE	BioLegend
**CD11c**	B-Ly6	PE Cy7	BD
**CD16**	3G8	AF 647	BioLegend
**CD14**	63D3	AF 700	BioLegend
**CD3**	UCHT1	APC FIRE750	BioLegend
**KLRG1**	13F12F2	APC	invitrogen
**HLA DR**	G46-6	BV421	BD
**CD45**	HI30	BV510	BioLegend
**Ki67**	B56	PerCpCy5.5	BD
**IFNγ**	B27	PE	BioLegend
**IL-17A**	N49-653	BV421	BD
**Perforin**	B-D48	PE Cy7	BioLegend
**Foxp3**	206D	BV421	BioLegend
**T-Bet**	O4-46	PE-CF594	BD
**TCR Vδ1**	REA173	FITC	Miltenyi Biotec
**CD27**	M-T271	PE-CF594	BD
**CD45RA**	HI100	PE Cy7	BioLegend
**TCRVγ9**	REA470	APC	Miltenyi Biotec
**Vδ2**	B6	APC Vio770	BioLegend
**TCRγδ**	REA591	APC	Miltenyi Biotec
**CD57**	NK-1	BV421	BD
**CX3CR1**	2A9-1	PE	BioLegend
**Perforin**	B-D48	PE Cy5.5	BioLegend

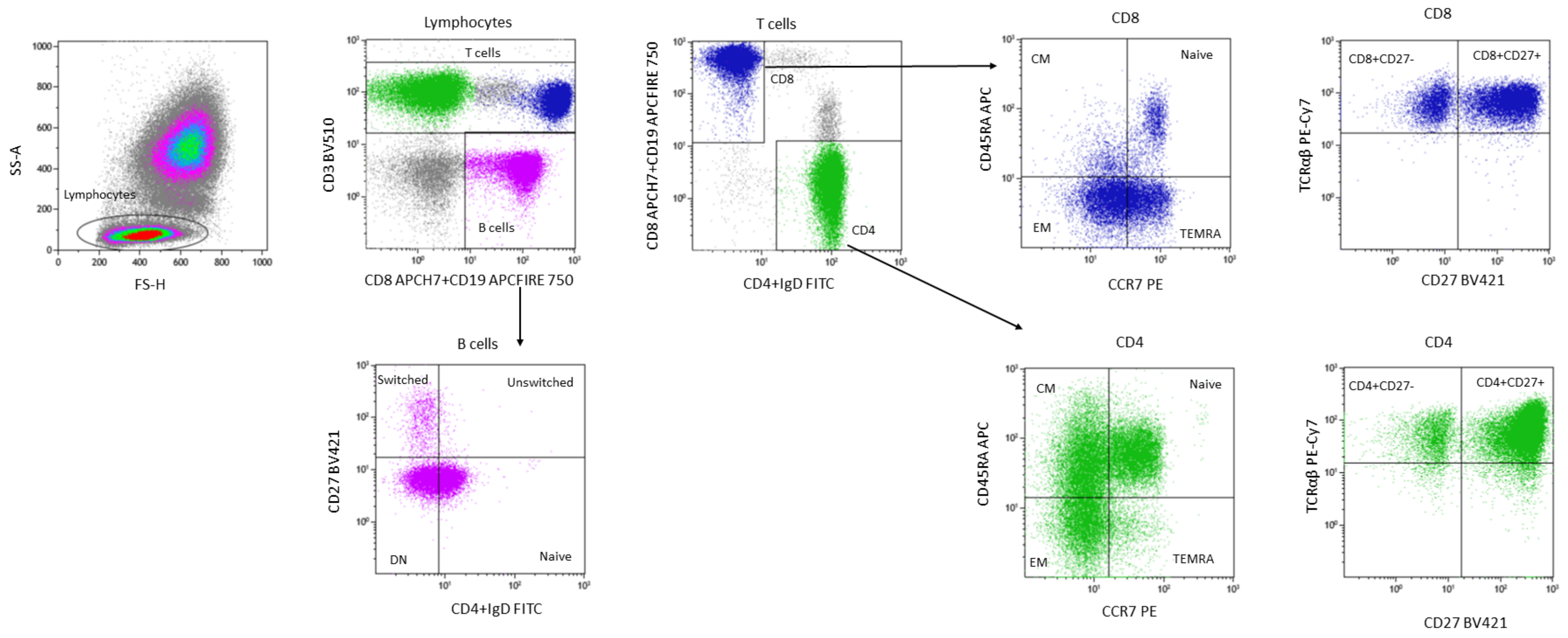

Supplementary figure 4: Gating strategy for phenotypic characterisation of T and B cells.

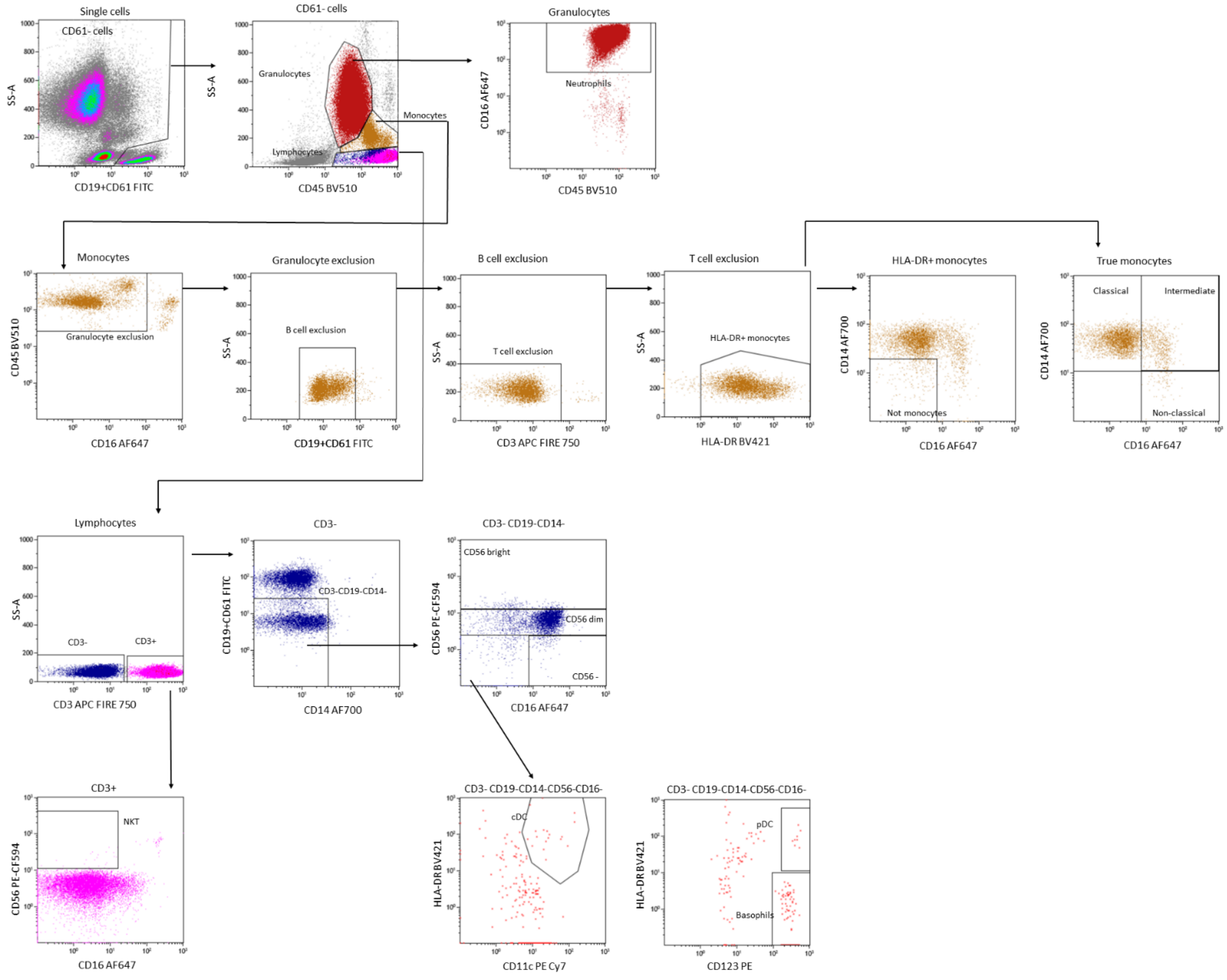

Supplementary figure 5: Gating strategy for phenotypic characterisation of innate immune cells.

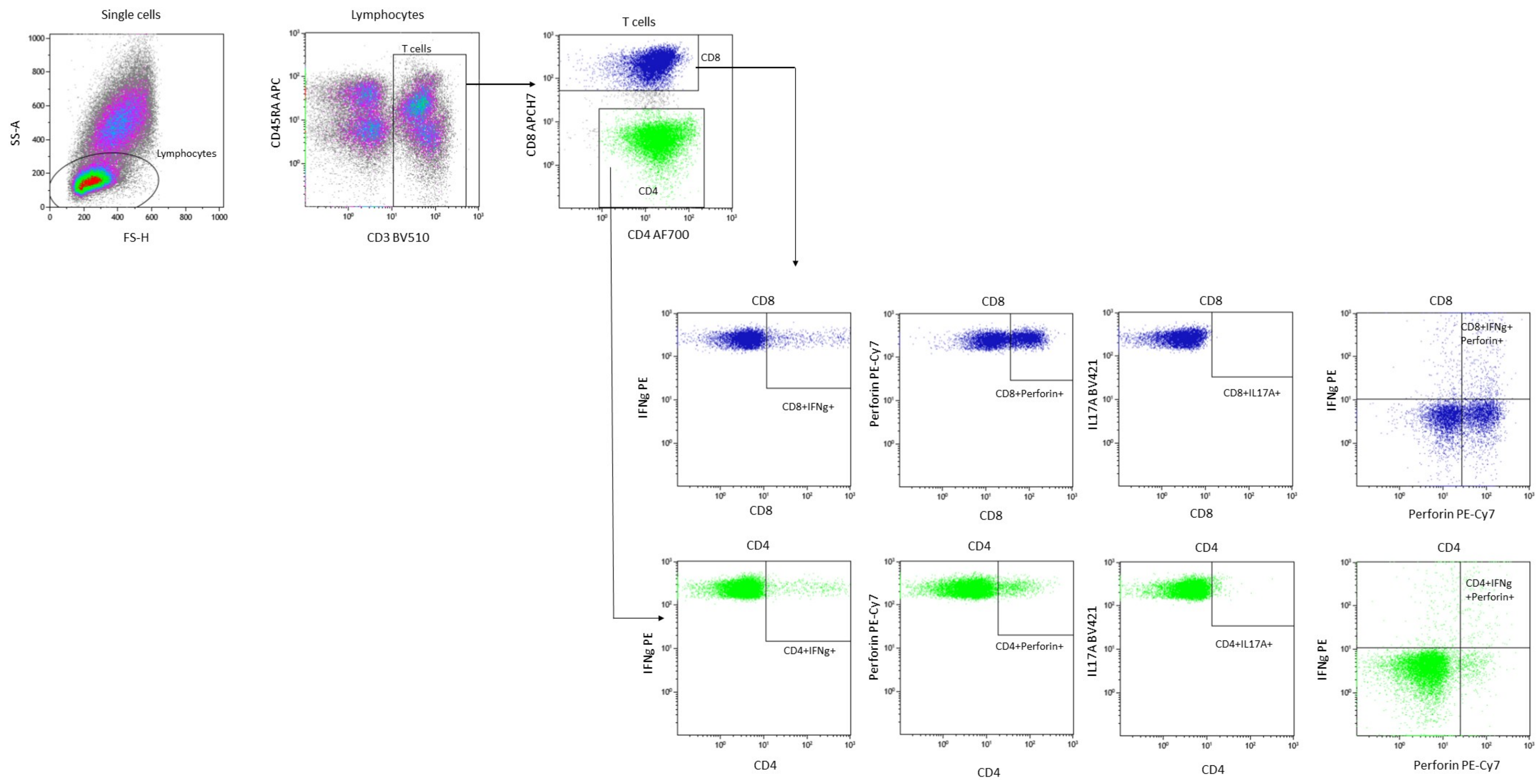

Supplementary figure 6: Gating strategy for phenotypic characterisation of proinflammatory and cytotoxic T cells from PBMC.

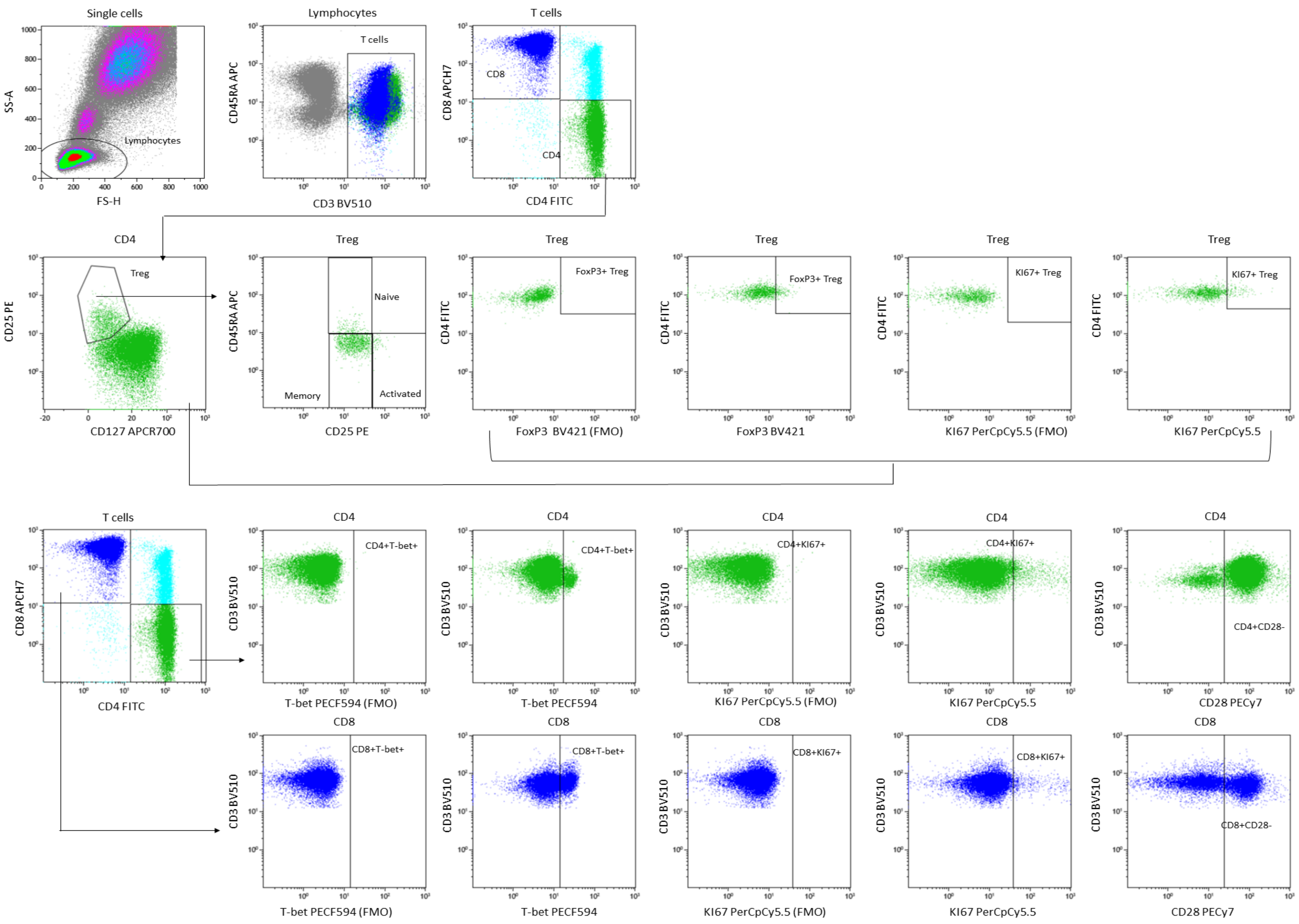

Supplementary figure 7: Gating strategy for phenotypic and proliferative characterisation of regulatory T cells (top panels) and effector T cells (bottom panels).

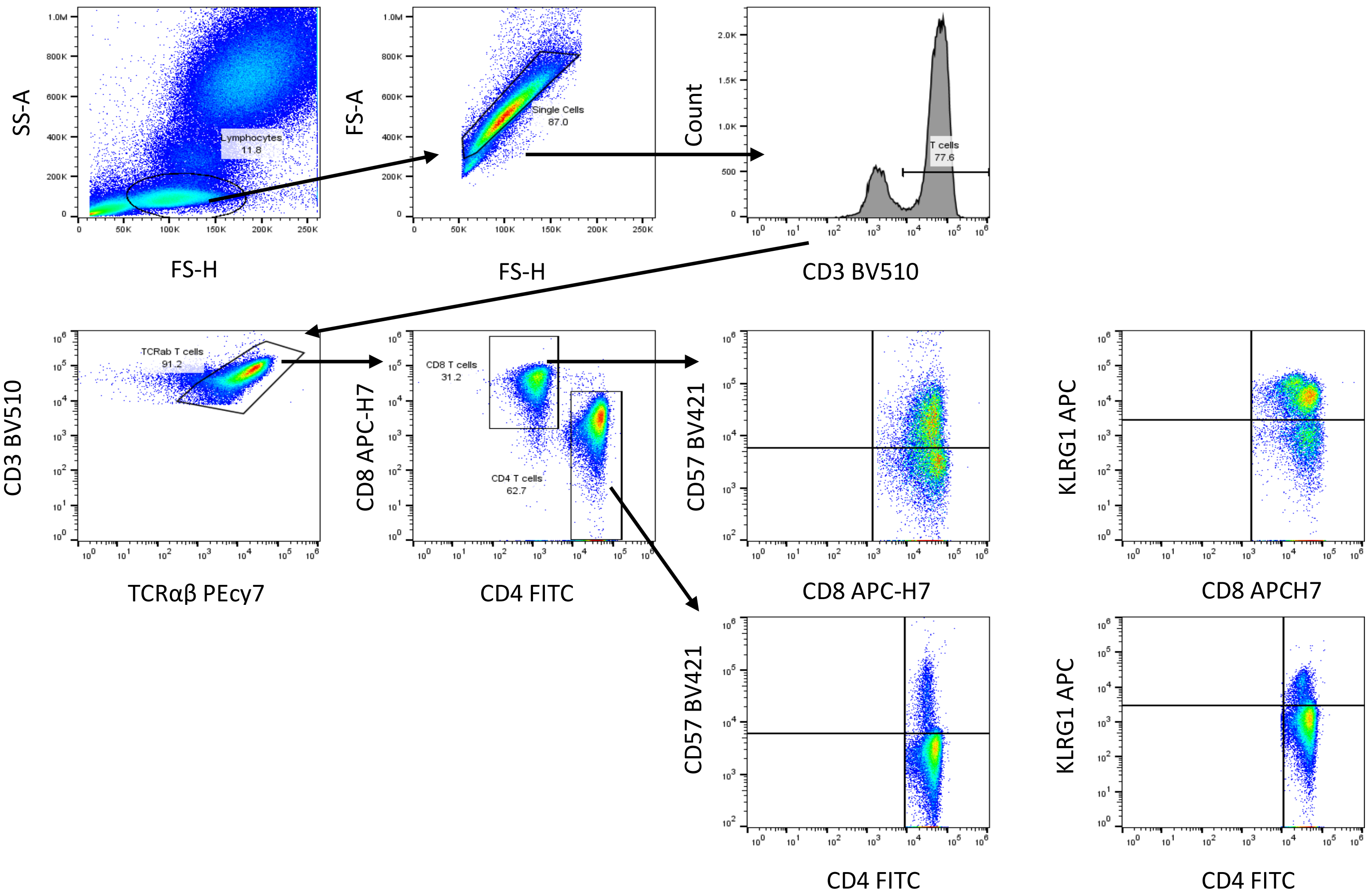

Supplementary figure 8: Gating strategy for phenotypic characterisation of highly differentiated T cells from whole blood.

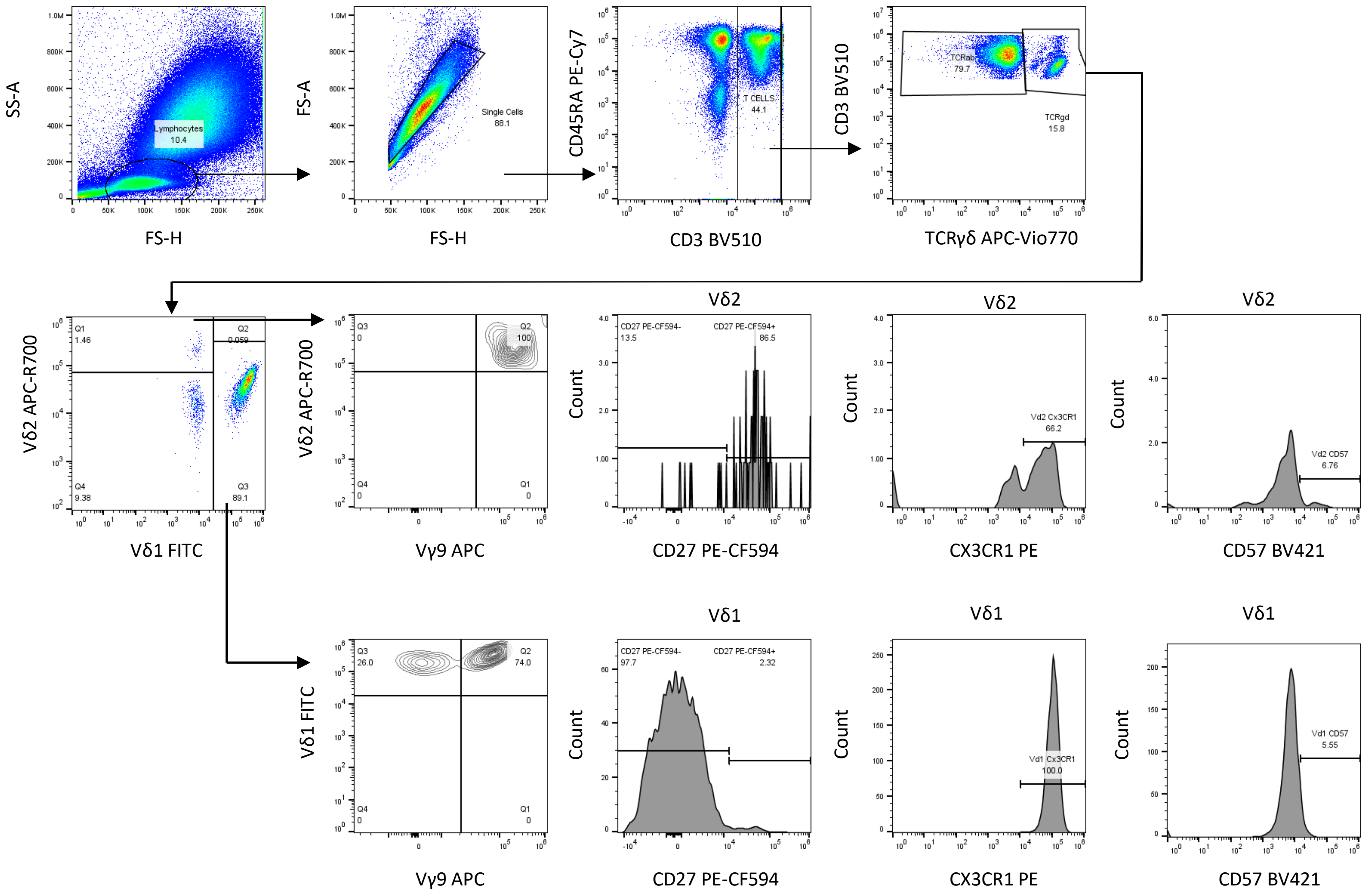

Supplementary figure 9. Gating strategy for phenotypic characterisation of gamma-delta T cells.

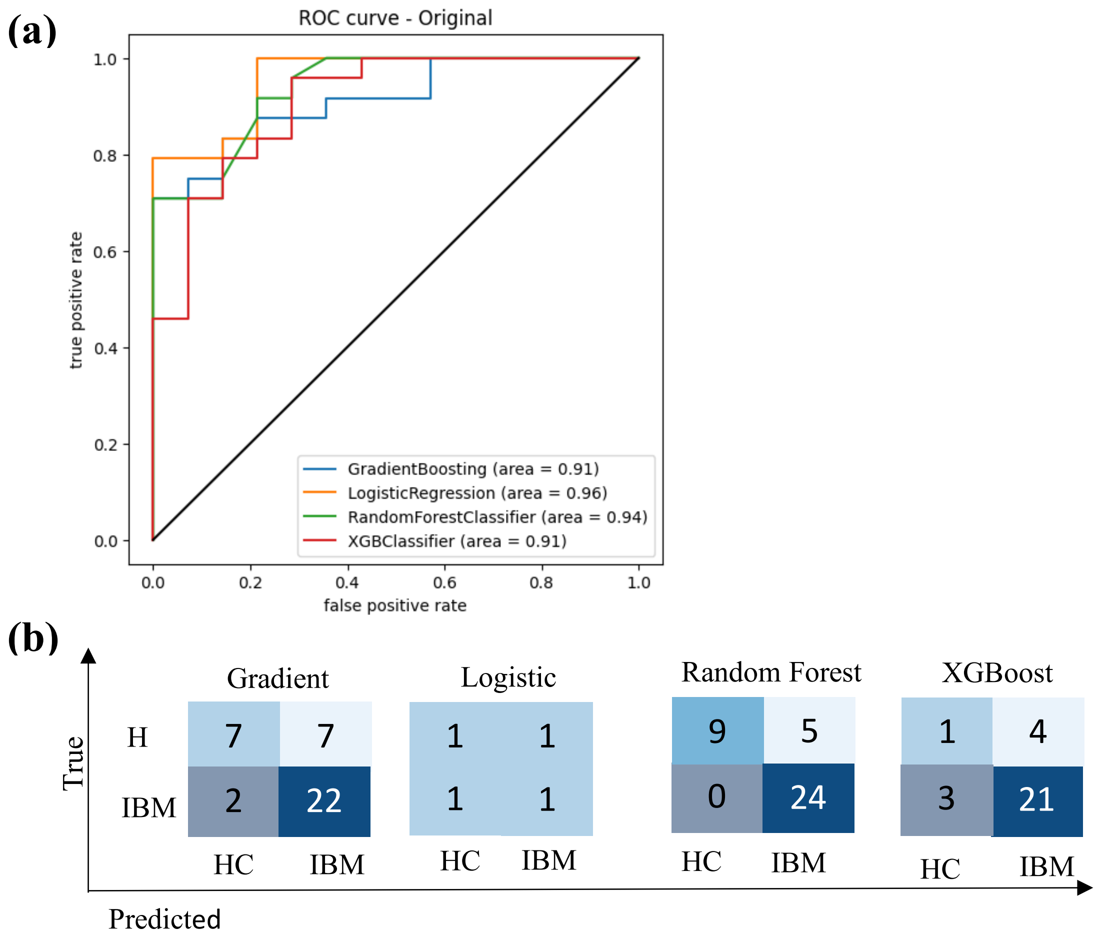

Supplementary figure 10: Machine learning algorithms tested in the study to identify best predicting algorithm. (a) Receiver operating characteristic curve analyses of three machine learning classifiers. **(b)** Confusion matrix per class (HC or IBM) for each algorithm.

Supplementary table 4: Performance comparison of three different machine learning models.

Model	Matthews Coefficient	AUROC	Precision score	Recall score	F1 score
**Gradient Boosting**	0.47	0.91	0.75	0.92	0.83
**Logistic Regression**	0.61	0.96	0.87	0.83	0.85
**Random Forrest**	0.72	0.94	0.82	1.0	0.90
**XGB Classifier**	0.6	0.91	0.84	0.86	0.85

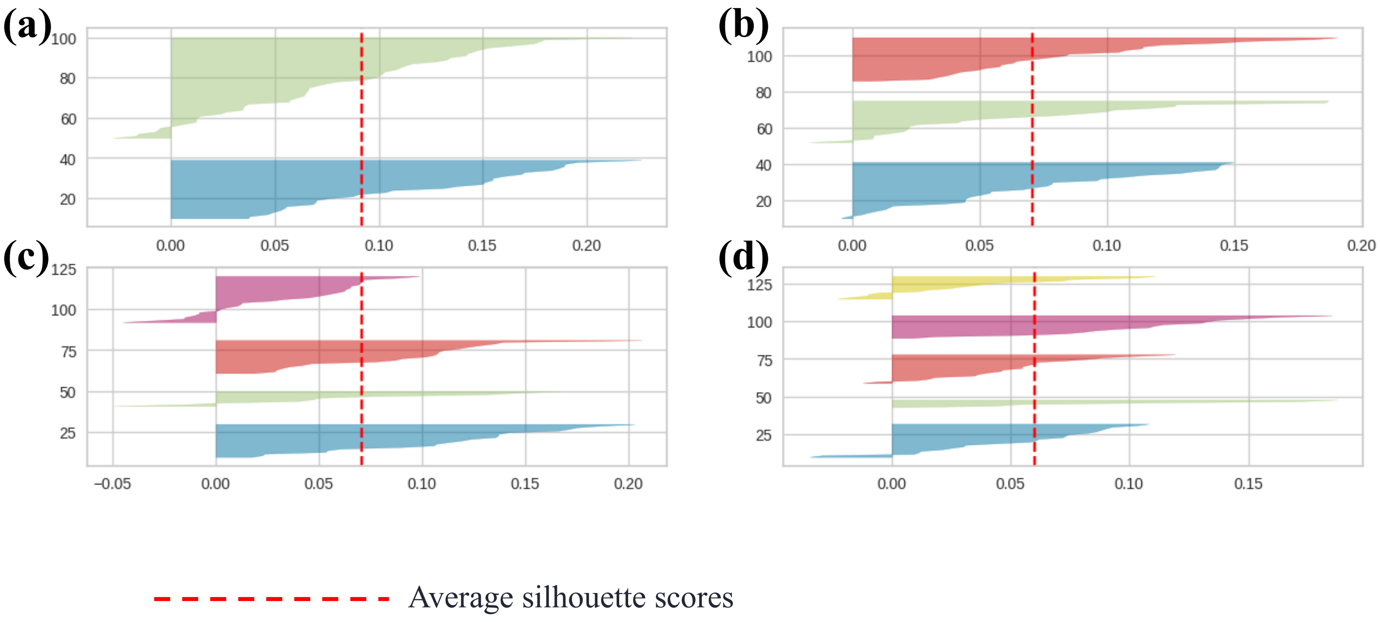

Supplementary figure 11. Silhouette Analysis for 2, 3, 4, and 5 clusters performed on the IBM dataset. The silhouette plots represent the separation distance between the resulting clusters based on K means clustering. We evaluated **(a)** two clusters, **(b)** three clusters, **(c)** four clusters and **(d)** five clusters. The average scores are indicated by the red-dotted vertical line.

Supplementary table 5: Determination of data distribution within immune cell variables using the Shapiro-wilk normality test.

**Parameter**	**W statistic**	***P*-value**	**Normality**
TCRγδ	0.740707	$1.25x10^{-13}$	Not Normal
Vδ2	0.900404	$1.17x10^{-7}$	Not Normal
Vγ9+Vδ2+	0.820995	$4.39x10^{-11}$	Not Normal
Vδ2+CD27-	0.878955	$1.03x10^{-8}$	Not Normal
Vδ2+Cx3Cr1+	0.945908	$7.28x10^{-5}$	Not Normal
Vδ2+CD57+	0.656531	$9.30x10^{-16}$	Not Normal
Vδ1+	0.942919	$4.44x10^{-5}$	Not Normal
Vγ9+Vδ1+	0.732581	$7.44x10^{-14}$	Not Normal
Vδ1+CD27-	0.910662	$4.21x10^{-7}$	Not Normal
Vδ1+Cx3Cr1+	0.886913	$2.45\ x10^{-8}$	Not Normal
Vδ1+CD57+	0.8732	$5.58\ x10^{-9}$	Not Normal
Vδ1-Vδ2-	0.899104	$1.00\ x10^{-7}$	Not Normal
CD4+	0.98861	0.38257	Normal
CD4Naive	0.966115	0.003001	Not Normal
CD4CM	0.986368	0.241217	Normal
CD4EM	0.955259	0.000373	Not Normal
CD4Temra	0.706341	$1.50\ x10^{-14}$	Not Normal
CD4+CD57+	0.74442	$1.59\ x10^{-13}$	Not Normal
CD4+KLRG1	0.867288	$3.04\ x10^{-9}$	Not Normal
CD4+CD27-	0.780528	$1.90\ x10^{-12}$	Not Normal
CD4+CD28-	0.7278	$5.51\ x10^{-14}$	Not Normal
CD4+KI67+	0.957894	0.000607	Not Normal
CD4+Tbet+	0.747313	$1.92\ x10^{-13}$	Not Normal
CD4+IFNγ+	0.891165	$3.96\ x10^{-8}$	Not Normal
CD4+Perf+	0.799459	$7.83\ x10^{-12}$	Not Normal
CD4+IL17A+	0.670682	$1.99\ x10^{-15}$	Not Normal
CD4+IFNγ+Perf+	0.758267	$4.00\ x10^{-13}$	Not Normal
CD8	0.977417	0.033162	Not Normal
CD8Naive	0.786609	$2.97\ x10^{-12}$	Not Normal
CD8CM	0.782225	$2.15\ x10^{-12}$	Not Normal
CD8EM	0.994115	0.881864	Normal
CD8Temra	0.973435	0.013866	Not Normal
CD8+CD27-	0.947573	$9.65\ x10^{-5}$	Not Normal
CD8+CD28-	0.954	0.000297	Not Normal
CD8+KI67+	0.639902	$3.92\ x10^{-16}$	Not Normal
CD8+Tbet+	0.95215	0.000213	Not Normal
CD8+IFNγ+	0.947492	$9.52\ x10^{-5}$	Not Normal
CD8+Perf+	0.964568	0.0022	Not Normal
CD8+IL17A+	0.730792	$6.65\ x10^{-14}$	Not Normal
CD8+IFNγ+Perf+	0.880331	$1.19\ x10^{-8}$	Not Normal
Bcells	0.98068	0.068681	Normal
DN_Bcells	0.971568	0.009296	Not Normal
NaiveBcells	0.973669	0.014585	Not Normal
NonSM	0.800956	$8.79\ x10^{-12}$	Not Normal

SM	0.898456	9.29×10^{-8}	Not Normal
Treg	0.960459	0.000986	Not Normal
TregFoxP3+	0.931408	7.40×10^{-6}	Not Normal
TregKI67+	0.960401	0.000975	Not Normal
NaiveTreg	0.728936	5.92×10^{-14}	Not Normal
ActTreg	0.907523	2.82×10^{-7}	Not Normal
Neutrophils	0.934748	1.22×10^{-5}	Not Normal
NKT	0.991499	0.639987	Normal
pDC	0.486342	4.21×10^{-19}	Not Normal
monocytes	0.758339	4.02×10^{-13}	Not Normal
Nkcells	0.968027	0.004433	Not Normal
Basophils	0.820175	4.10×10^{-11}	Not Normal
cDC	0.89226	4.49×10^{-8}	Not Normal
CD56-16b	0.664315	1.41×10^{-15}	Not Normal
CD56b	0.904719	1.99×10^{-7}	Not Normal
CD56dim	0.549791	5.73×10^{-18}	Not Normal
classical_monocytes	0.929153	5.30×10^{-6}	Not Normal
int_monocytes	0.919712	1.40×10^{-6}	Not Normal
inflam_monocytes	0.929232	5.36×10^{-6}	Not Normal

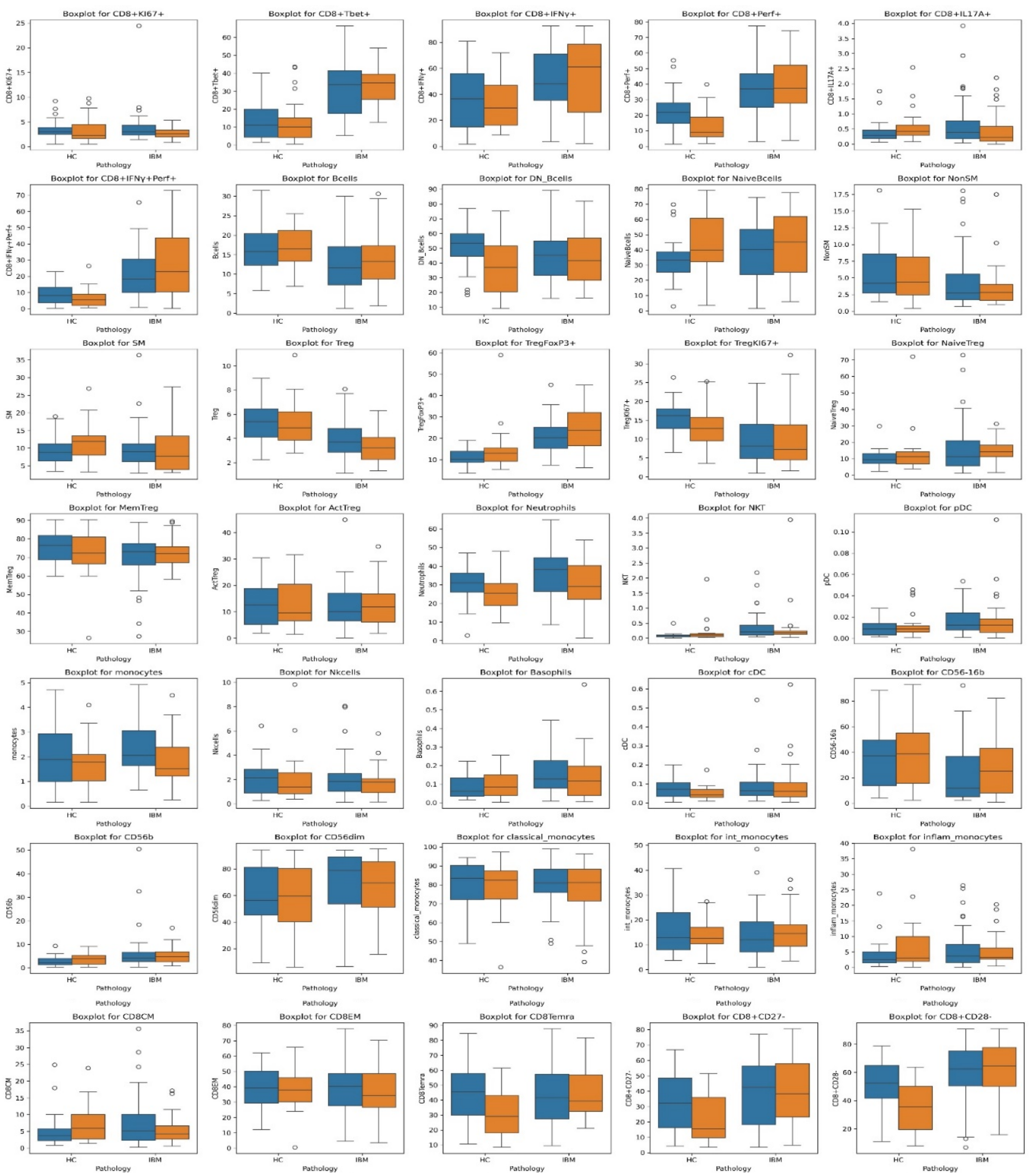

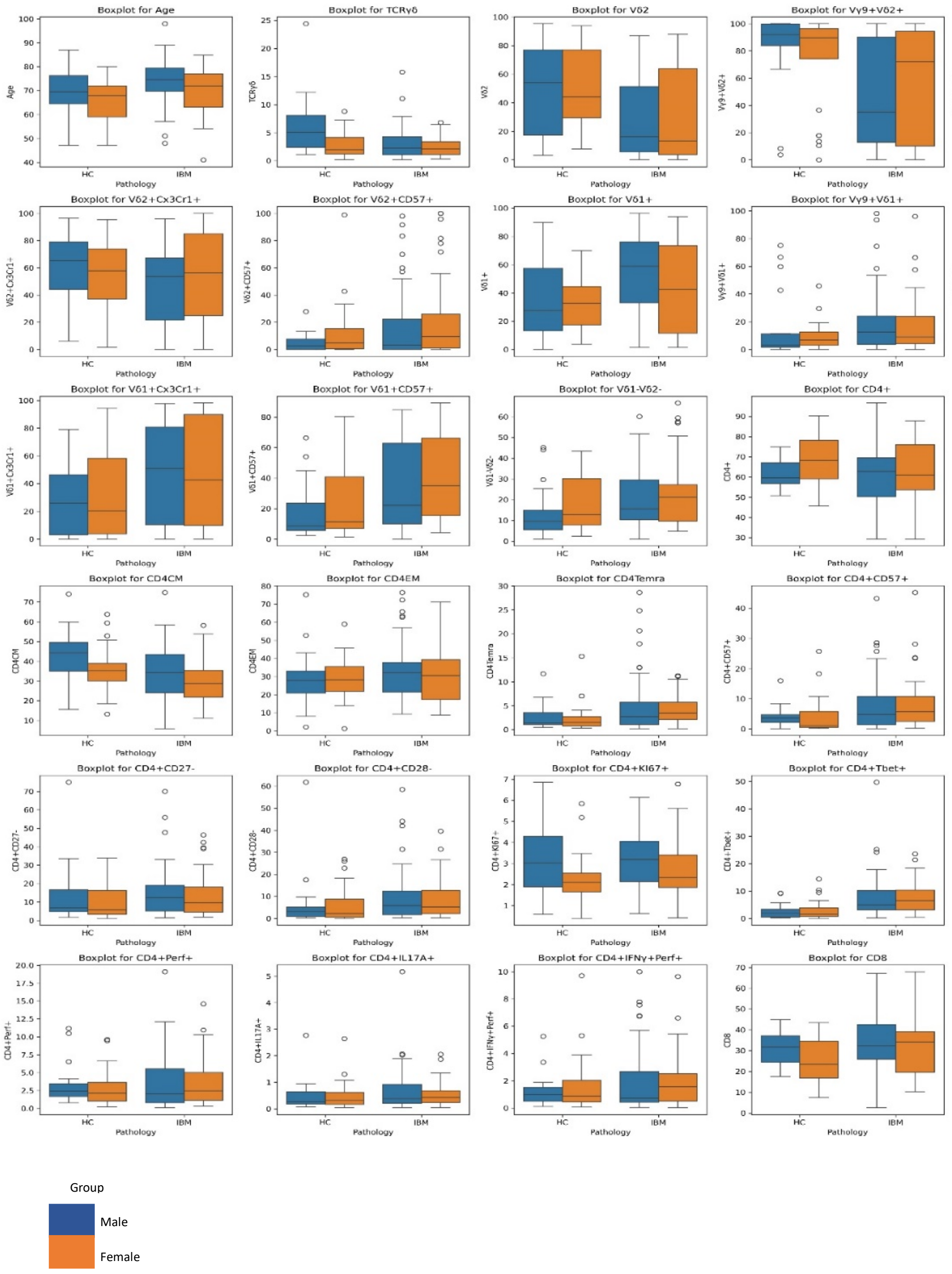

Supplementary Figure 12: Comparison of age and immune cell populations in IBM and HC stratified based on gender (Male represented as blue boxes vs Female represented as orange boxes). The statistical analysis was performed using Kruskal-Wallis ANOVA with Dunn's post hoc comparison.

Supplementary table 6: influence of biological sex on immune cell population and pathology group. Kruskal-Wallis ANOVA with Dunn's post hoc comparison. Only populations that passed the Kruskal-Wallis test are shown.

	Kruskal-Wallis H-statistic	*P*-value	Dunns post hoc results
TCRγδ	12.78	0.01	HC_M HC_F IBM_M IBM_F<br>HC_M 1.000000 0.038180 0.011201 0.005366<br>HC_F 0.038180 1.00000 1.000000 1.000000<br>IBM_M 0.011201 1.00000 1.000000 1.000000<br>IBM_F 0.005366 1.00000 1.000000 1.000000
Vδ2	14.30	2.52E-03	HC_M HC_F IBM_M IBM_F<br>HC_M 1.000000 1.000000 0.038837 0.136840<br>HC_F 1.000000 1.000000 0.016482 0.077541<br>IBM_M 0.038837 0.016482 1.000000 1.000000<br>IBM_F 0.136840 0.077541 1.000000 1.000000
Vγ9+Vδ2+	12.48	0.01	HC_M HC_F IBM_M IBM_F<br>HC_M 1.000000 1.000000 0.013089 0.240515<br>HC_F 1.000000 1.000000 0.063486 0.807948<br>IBM_M 0.013089 0.063486 1.000000 1.000000<br>IBM_F 0.240515 0.807948 1.000000 1.000000
Vδ1+	8.77	0.03	HC_M HC_F IBM_M IBM_F<br>HC_M 1. 00000 1.000000 0.201220 1.000000<br>HC_F 1. 00000 1.000000 0.051136 1.000000<br>IBM_M 0.20122 0.051136 1.000000 1.000000<br>IBM_F 1.00000 1.000000 1.000000 1.000000
Vδ1+CD57+	13.15	4.32E-03	HC_M HC_F IBM_M IBM_F<br>HC_M 1.000000 1.000000 0.080703 0.013370<br>HC_F 1.000000 1.000000 0.319324 0.058334<br>IBM_M 0.080703 0.319324 1.000000 1.000000<br>IBM_F 0.013370 0.058334 1.000000 1.000000
CD4 CM	11.31	0.01	HC_M HC_F IBM_M IBM_F<br>HC_M 1.000000 0.855924 0.152031 0.005875<br>HC_F 0.855924 1.000000 1.000000 0.373573<br>IBM_M 0.152031 1.000000 1.000000 0.803161<br>IBM_F 0.005875 0.373573 0.803161 1.000000
CD4 Temra	8.49	0.04	HC_M HC_F IBM_M IBM_F<br>HC_M 1.000000 1.000000 1.000000 0.306749<br>HC_F 1.000000 1.000000 0.277417 0.059410<br>IBM_M 1.000000 0.277417 1.000000 1.000000<br>IBM_F 0.306749 0.059410 1.000000 1.000000
CD4+CD28-	9.16	0.03	HC_M HC_F IBM_M IBM_F<br>HC_M 1.000000 1.000000 0.194255 0.208155<br>HC_F 1.000000 1.000000 0.181498 0.201550<br>IBM_M 0.194255 0.181498 1.000000 1.000000<br>IBM_F 0.208155 0.201550 1.000000 1.000000
CD4+KI67+	9.68	0.02	HC_M HC_F IBM_M IBM_F<br>HC_M 1.000000 0.318492 1.000000 1.000000

			HC_F 0.318492 1.000000 0.018986 1.000000<br>IBM_M 1.000000 0.018986 1.000000 0.423006<br>IBM_F 1.000000 1.000000 0.423006 1.000000
CD4+Tbet+	31.61	6.33E-07	HC_M HC_F IBM_M IBM_F<br>HC_M 1.000000 1.000000 0.000442 0.000735<br>HC_F 1.000000 1.000000 0.000246 0.000485<br>IBM_M 0.000442 0.000246 1.000000 1.000000<br>IBM_F 0.000735 0.000485 1.000000 1.000000
CD8 Naive	16.16	1.05E-03	HC_M HC_F IBM_M IBM_F<br>HC_M 1.000000 0.037975 1.000000 1.000000<br>HC_F 0.037975 1.000000 0.000474 0.111501<br>IBM_M 1.000000 0.000474 1.000000 0.733524<br>IBM_F 1.000000 0.111501 0.733524 1.000000
CD8 Temra	10.05	0.02	HC_M HC_F IBM_M IBM_F<br>HC_M 1.000000 0.087691 1.000000 1.000000<br>HC_F 0.087691 1.000000 0.065942 0.023117<br>IBM_M 1.000000 0.065942 1.000000 1.000000<br>IBM_F 1.000000 0.023117 1.000000 1.000000
CD8+CD27-	13.32	3.99E-03	HC_M HC_F IBM_M IBM_F<br>HC_M 1.00000 0.337810 1.000000 1.000000<br>HC_F 0.33781 1.000000 0.005314 0.008032<br>IBM_M 1.00000 0.005314 1.000000 1.000000<br>IBM_F 1.00000 0.008032 1.000000 1.000000
CD8+CD28-	25.43	1.25E-05	HC_M HC_F IBM_M IBM_F<br>HC_M 1.000000 0.150173 0.942807 0.282096<br>HC_F 0.150173 1.000000 0.000127 0.000019<br>IBM_M 0.942807 0.000127 1.000000 1.000000<br>IBM_F 0.282096 0.000019 1.000000 1.000000
CD8+Tbet+	39.64	1.27E-08	HC_M HC_F IBM_M IBM_F<br>HC_M 1.000000 1.000000 0.000242 0.000081<br>HC_F 1.000000 1.000000 0.000034 0.000012<br>IBM_M 0.000242 0.000034 1.000000 1.000000<br>IBM_F 0.000081 0.000012 1.000000 1.000000
CD8+IFNγ+	11.38	9.85E-03	HC_M HC_F IBM_M IBM_F<br>HC_M 1.000000 1.000000 0.71850 0.308848<br>HC_F 1.000000 1.000000 0.05219 0.018439<br>IBM_M 0.718500 0.052190 1.00000 1.000000<br>IBM_F 0.308848 0.018439 1.00000 1.000000
CD8+Perf+	38.35	2.38E-08	HC_M HC_F IBM_M IBM_F<br>HC_M 1.000000 0.252968 0.063291 0.020159<br>HC_F 0.252968 1.000000 0.000001 3.317281e-07<br>IBM_M 0.063291 1.011608e-06 1.000000 1.000000<br>IBM_F 0.020159 3.317281e-07 1.000000 1.000000
CD8+IFNγ+Perf+	30.80	9.35E-07	HC_M HC_F IBM_M IBM_F<br>HC_M 1.000000 1.000000 0.008584 0.002341<br>HC_F 1.000000 1.000000 0.000156 0.000040<br>IBM_M 0.008584 0.000156 1.000000 1.000000<br>IBM_F 0.002341 0.000040 1.000000 1.000000
B cells	10.85	0.01	HC_M HC_F IBM_M IBM_F<br>HC_M 1.000000 1.000000 0.136435 0.946832

			HC_F 1.000000 1.000000 0.021548 0.320138<br>IBM_M 0.136435 0.021548 1.000000 1.000000<br>IBM_F 0.946832 0.320138 1.000000 1.000000
Treg	26.72	6.73E-06	HC_M HC_F IBM_M IBM_F<br>HC_M 1.000000 1.000000 0.018033 0.000269<br>HC_F 1.000000 1.000000 0.015980 0.000176<br>IBM_M 0.018033 0.015980 1.000000 0.628544<br>IBM_F 0.000269 0.000176 0.628544 1.000000
TregFoxP3+	41.85	4.32E-09	HC_M HC_F IBM_M IBM_F<br>HC_M 1.000000e+00 1.000000 0.000029 9.781076e-07<br>HC_F 1.000000e+00 1.000000 0.000890 3.396302e-05<br>IBM_M 2.918252e-05 0.000890 1.000000 1.000000e+00<br>IBM_F 9.781076e-07 0.000034 1.000000 1.000000e+00
Treg KI67+	16.51	0.0008	HC_M HC_F IBM_M IBM_F<br>HC_M 1.000000 0.629903 0.002413 0.002835<br>HC_F 0.629903 1.000000 0.388847 0.343042<br>IBM_M 0.002413 0.388847 1.000000 1.000000<br>IBM_F 0.002835 0.343042 1.000000 1.000000
Neutrophils	11.61	0.008	HC_M HC_F IBM_M IBM_F<br>HC_M 1.000000 1.00000 0.468812 1.000000<br>HC_F 1.000000 1.00000 0.006610 1.000000<br>IBM_M 0.468812 0.00661 1.000000 0.305612<br>IBM_F 1.000000 1.00000 0.305612 1.000000
NKT cells	24.93	1.59E-05	HC_M HC_F IBM_M IBM_F<br>HC_M 1.000000 0.909377 0.000050 0.001971<br>HC_F 0.909377 1.000000 0.013095 0.160065<br>IBM _M 0.000050 0.013095 1.000000 1.000000<br>IBM_F 0.001971 0.160065 1.000000 1.000000
Basophils	9.65	0.02	HC_M HC_F IBM_M IBM_F<br>HC_M 1.000000 1.000000 0.045996 1.000000<br>HC_F 1.000000 1.000000 0.110677 1.000000<br>IBM_M 0.045996 0.110677 1.000000 0.959059<br>IBM_F 1.000000 1.000000 0.959059 1.000000
CD56-16b+	11.76	0.008	HC_M HC_F IBM_M IBM_F<br>HC_M 1.000000 1.000000 0.121831 1.000000<br>HC_F 1.000000 1.000000 0.011510 0.338161<br>IBM_M 0.121831 0.011510 1.000000 1.000000<br>IBM_F 1.000000 0.338161 1.000000 1.000000
CD56b	9.18	0.02	HC_M HC_F IBM_M IBM_F<br>HC_M 1.000000 1.000000 0.062669 0.033960<br>HC_F 1.000000 1.000000 1.000000 0.949479<br>IBM_M 0.062669 1.000000 1.000000 1.000000<br>IBM_F 0.033960 0.949479 1.000000 1.000000
CD56dim	8.70	0.03	HC_M HC_F IBM_M IBM_F<br>HC_M 1.000000 1.000000 0.314936 1.000000<br>HC_F 1.000000 1.000000 0.040403 0.645809<br>IBM_M 0.314936 0.040403 1.000000 1.000000<br>IBM_F 1.000000 0.645809 1.000000 1.000000

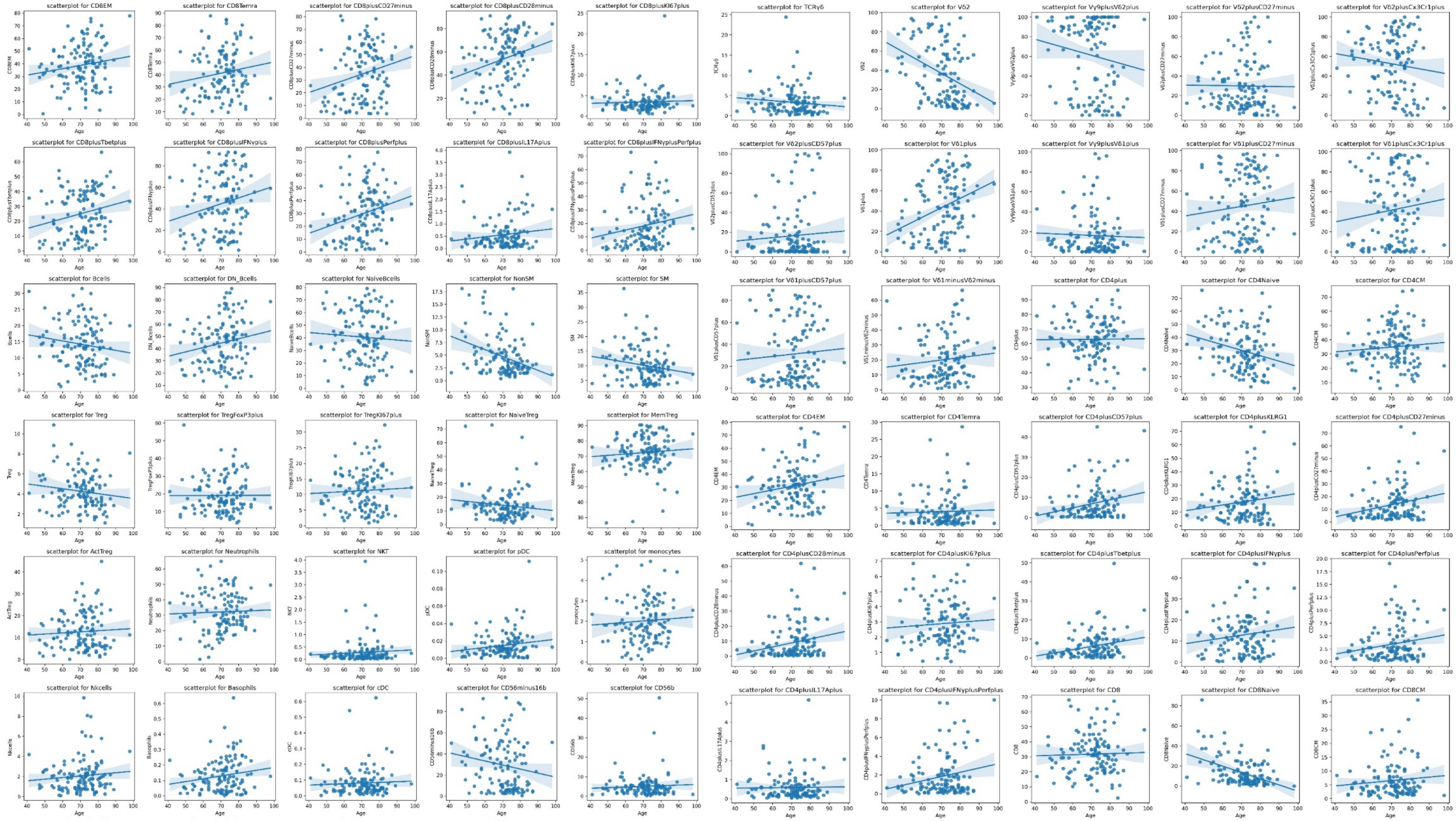

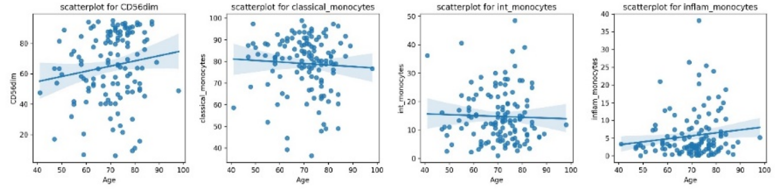

Supplementary figure 13: Scatter plots to determine linear relationship between age and immune cell populations. Data are pooled from both IBM and HC.

Supplementary table 7: Linear regression analysis between age and parameter (immune cell population). VIF= variable inflation factor.

Parameter	VIF	R-squared	Age *P*-value	Intercept
TCRγδ	1	0.02	0.14	4.87
Vδ2	1	0.14	0.00	113.55
Vγ9+Vδ2+	1	0.02	0.11	99.19
Vδ2+CD27-	1	0.02	0.17	43.25
Vδ2+Cx3Cr1+	1	0.01	0.18	76.79
Vδ2+CD57+	1	8.81E-03	0.32	17.97
Vδ1+	1	0.11	0.00	-21.16
Vγ9+Vδ1+	1	6.40E-03	0.39	19.49
Vδ1+CD27-	1	0.01	0.26	22.78
Vδ1+Cx3Cr1+	1	0.01	0.20	14.06
Vδ1+CD57+	1	0.01	0.21	9.39
Vδ1-Vδ2-	1	8.43E-03	0.32	9.81
CD4+	1	6.35E-04	0.79	66.27
CD4Naive	1	0.08	0.00	60.11
CD4CM	1	1.35E-03	0.69	31.14
CD4EM	1	1.09E-03	0.72	25.79
CD4Temra	1	2.43E-03	0.59	4.41
CD4+CD57+	1	0.01	0.27	1.35
CD4+KLRG1	1	2.54E-06	0.99	15.33
CD4+CD27-	1	0.02	0.08	1.30
CD4+CD28-	1	0.03	0.07	-2.10
CD4+KI67+	1	0.01	0.25	1.82
CD4+Tbet+	1	0.03	0.07	-0.13
CD4+IFNγ+	1	2.70E-03	0.58	13.57
CD4+Perf+	1	0.05	0.02	-0.96
CD4+IL17A+	1	4.37E-03	0.47	0.66
CD4+IFNγ+Perf+	1	7.23E-03	0.36	0.58
CD8	1	3.92E-03	0.50	25.44
CD8Naive	1	0.09	0.00	33.23
CD8CM	1	8.39E-03	0.32	2.93
CD8EM	1	5.70E-03	0.41	31.34
CD8Temra	1	0.03	0.08	20.59
CD8+CD27-	1	0.08	0.00	-5.43
CD8+CD28-	1	0.06	0.00	16.71
CD8+KI67+	1	1.10E-04	0.91	2.97
CD8+Tbet+	1	0.04	0.03	4.09
CD8+IFNγ+	1	0.04	0.02	6.58
CD8+Perf+	1	0.08	0.00	-5.18
CD8+IL17A+	1	0.05	0.01	-0.23
CD8+IFNγ+Perf+	1	0.05	0.02	-4.38
Bcells	1	0.01	0.25	18.38
DN_Bcells	1	0.03	0.04	21.33
NaiveBcells	1	3.73E-03	0.50	48.98
NonSM	1	0.05	0.01	8.14
SM	1	0.02	0.09	14.43
Treg	1	0.02	0.17	5.42

TregFoxP3+	1	2.70E-03	0.57	15.12
TregKI67+	1	8.10E-04	0.76	11.56
NaiveTreg	1	0.03	0.05	20.96
ActTreg	1	3.76334E-05	0.95	12.04
Neutrophils	1	1.63E-03	0.66	28.71
NKT	1	0.02	0.11	-0.01
pDC	1	1.13E-03	0.71	0.01
monocytes	1	8.02E-03	0.33	1.33
Nkcells	1	1.58E-03	0.67	1.48
Basophils	1	0.02	0.10	0.03
cDC	1	1.91E-03	0.63	0.08
CD56-16b	1	0.04	0.03	54.54
CD56b	1	4.77E-04	0.81	4.61
CD56dim	1	0.03	0.05	42.66
classical_monocytes	1	3.12E-03	0.55	85.06
int_monocytes	1	3.50188E-05	0.95	13.43
inflam_monocytes	1	0.02	0.12	0.53
